# Conserved and divergent mitochondrial assemblies in kinetoplastid parasites

**DOI:** 10.1016/j.molcel.2026.08.031

**Published:** 2026-10-01

**Authors:** Md Solayman, Yi-Ting Liao, Sarah E. Calvo, Andres F. Vacas, Malvina Papanastasiou, Keith D. Rivera, DR Mani, Brian W. Boyle, Felicia G. Deng, MitoCarta Tree of Life Consortium, Cora N. Betsinger, Steven A. Carr, Michael Z. Chen, Manoj T. Duraisingh, Michelle Y. Fry, Manish Goyal, Tiana M. Issa, Minsoo Kim, Cameron Genxuan Lian, Bridget E. Luce, Syed Yusuf Mian, Khanh Nguyen, Aditya S. Paul, John Samuelson, Jonathan A. Stefely, Namrata D. Udeshi, Vamsi K. Mootha, Inna Aphasizheva, Luke H. Chao, Ruslan Aphasizhev

**Affiliations:** 1 Department of Molecular and Cell Biology, Boston University Medical Campus, Boston, MA 02118, USA; 2 Department of Molecular Biology, Massachusetts General Hospital, Boston, MA 02114, USA; 3 Department of Genetics, Harvard Medical School, Boston, MA 02115, USA; 4 Department of Systems Biology, Harvard Medical School, Boston, MA 02115, USA; 5 Broad Institute of MIT and Harvard, Cambridge, MA 02142, USA; 6 Howard Hughes Medical Institute, Massachusetts General Hospital, Boston, MA 02114, USA; 7 These authors contributed equally; 8 Lead contact

**Keywords:** Kinetoplastids, *Trypanosoma*, *Leishmania*, mitochondria, complexome profiling, respiratory complexes, RNA editing, mass spectrometry, cryo-EM

## Abstract

The mitochondrial proteomes of *Leishmania tarentolae* and *Trypanosoma brucei* contain ~1,700 proteins, most of which lack homologs in higher eukaryotes. Here, we integrate complexome profiling and cryo-electron microscopy to define conserved and lineage-specific macromolecular assemblies that support mitochondrial functions in these kinetoplastid protozoa. Comparative analyses reveal species- and life-stage-dependent differences in the abundance and composition of respiratory, metabolic, RNA-processing, and other complexes, refining and expanding current annotations. Structures of *L. tarentolae* respiratory complexes CIII_2_, CIV_2_, and CV identify nine previously unrecognized nuclear-encoded subunits and resolve five mitochondrially encoded proteins, including products of pan-edited mRNAs. These reconstructions uncover architectural innovations: a QCR8 N-terminal extension that plugs the vestigial MPPα/β cavity in CIII_2_, a CIV_2_ dimer stabilized by an extensive clade-restricted interface, and a CV dimer containing the mitochondrially encoded ATP6 subunit. Together, our findings reveal how kinetoplastids assemble specialized mitochondrial machineries while incorporating diverged components into core modules shared across eukaryotes.

## In brief

Complexome profiling integrated with single-particle cryo-EM reveals how conserved and lineage-specific mitochondrial proteins assemble into macromolecular complexes in the kinetoplastid protozoans *Trypanosoma brucei* and *Leishmania tarentolae*.

## Graphical Abstract

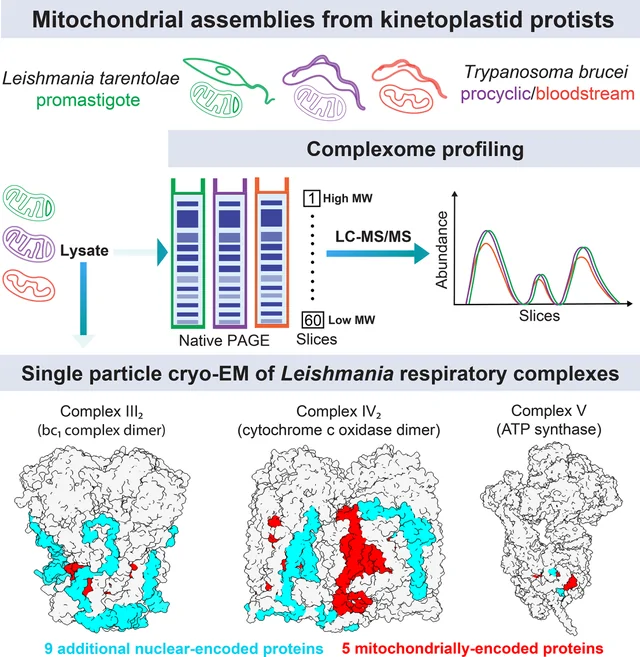

## INTRODUCTION

Mitochondria, which originated from an α-proteobacterial endosymbiont roughly two billion years ago, have diversified extensively, giving rise to lineage-specific macromolecular assemblies and specialized metabolic pathways.^1^ Among the most striking examples of such innovation are the exceptional genome architecture, RNA processing systems, and energy and metabolic strategies found in the class Kinetoplastea, an early-branching lineage within the Discoba clade.^2^

Kinetoplastid flagellated protozoans include medically and veterinarily important species, most notably parasites of the genera *Leishmania* and *Trypanosoma*. Their single mitochondrion contains a catenated DNA network composed of maxicircles, homologous to conventional mitochondrial genomes, and thousands of minicircles that encode guide RNAs (gRNAs) required for U-insertion and deletion editing of maxicircle-derived mRNAs. The kinetoplast DNA (kDNA) is compacted into a disk-shaped nucleoid positioned adjacent to the basal body and physically linked to the flagellum.

The unusually large complexes that nonetheless retain clear bacterial ancestry, such as the protein-rich mitoribosome,^3^ together with assemblies unique to kinetoplastids, including the RNA editing machinery,^4^ partially account for the substantial expansion of the mitoproteomes in *L. tarentolae* and *T. brucei*. Yet most of the ~1,700 nuclear-encoded mitoproteins, of which more than 50% lack detectable homologs in model eukaryotes, remain unassigned to functions, pathways, or complexes.^5^ Moreover, how these evolutionarily derived elements remodel ancient mitochondrial systems, such as the respiratory chain, remains largely unexplored.

Cryogenic electron microscopy (cryo-EM) reconstructions of the mitoribosome,^3,6^ RNA editing substrate binding complex (RESC)^7^ and RNA editing catalytic complex (RECC),^8^ metabolic enzymes 3-methylcrotonyl-CoA^9,10^ and propionyl-CoA carboxylases^11^, and ATP synthase^12^ have revealed pronounced parasite-specific features. Complementary affinity purification–mass spectrometry studies show that many mitochondrial processes, including transcription, RNA editing, polyadenylation, and translation, are coupled through interactions among intrinsically stable assemblies.^13–17^ Despite these advances, systems-level, high-resolution mitochondrial complexomes of divergent kinetoplastids across their vector and host stages remain lacking.

As part of the MitoCarta Tree of Life (Mito-TOL) consortium, we defined MitoCarta inventories for *L. tarentolae* and *T. brucei*.^5^ Building on these resources, we applied complexome profiling, combining blue-native polyacrylamide gel electrophoresis of mitochondrial lysates with mass spectrometry (BN-PAGE-MS), to determine how nuclear- and kDNA-encoded proteins assemble into complexes. Our analyses encompassed *L. tarentolae* promastigotes (LtPMF) and both procyclic insect-form (TbPCF) and long slender bloodstream-form (TbBSF) *T. brucei*. This approach assigned complex membership in agreement with published structures and revealed species- and developmental stage-specific differences in the size, abundance, and composition of mitochondrial assemblies. Furthermore, complexome profiling identified previously uncharacterized subunits within established complexes and refined existing annotations.

Extending the complexome profiling, we determined cryo-EM structures of the LtPMF respiratory complexes III_2_, IV_2_, and V, revealing extensive remodeling of the kinetoplastid electron transport chain. These near-atomic-resolution models identify additional nuclear-encoded subunits and resolve multiple proteins translated from kDNA-encoded mRNAs, including products of pan-edited transcripts. The LtPMF CIII_2_ structure reveals an N-terminal extension of QCR8 that plugs the cavity of the vestigial mitochondrial processing peptidase α/β heterodimer. The LtPMF CIV_2_ structure reveals an extensive kinetoplastid-specific dimerization interface, whereas the LtPMF CV dimer exhibits a conserved architecture also present in *T. brucei*.^12^ Together, these results provide systems-level insight into the organization of kinetoplastid mitochondrial complexes and establish a structural framework for rational therapeutic targeting.

## RESULTS

### Kinetoplastid mitochondrial complexomes

Mitochondria enriched from LtPMF, TbPCF, and TbBSF by isotonic lysis and OptiPrep density gradient centrifugation were solubilized with n-dodecyl-β-D-maltoside, cleared by centrifugation, and separated by BN-PAGE (Figure 1A). Gel lanes were sectioned into 60 uniform slices and each analyzed by LC–MS/MS, yielding 2,790, 2,126, and 2,094 protein groups in LtPMF, TbPCF, and TbBSF, respectively (Figure 1B; Table S1). Of these, 1,238, 1,228, and 1,027 mapped to the corresponding MitoCarta inventories^5^ (Figure 1C; Table S2).

To quantify the relative abundances of 20 previously annotated complexes comprising 454 proteins (Table S3), we integrated mass spectrometry data from whole-cell lysates^5^ with our BN-PAGE datasets. Across a dynamic range spanning four orders of magnitude, ATP synthase (CV) emerged as the most abundant complex, whereas the mitochondrial calcium uniporter (MCU) was the least abundant (Figure 1D). Metabolic and mitochondrial maintenance complexes were generally more abundant than those associated with gene expression. Notably, among the latter, the enigmatic RNA-binding KMRP1/2 tetramer^4,18,19^ was the most highly expressed. In TbBSF, complex III was markedly reduced, consistent with impaired editing of the kDNA-encoded cytochrome b mRNA,^20^ whereas depletion of complex IV reflects the bloodstream form’s reliance on glycolysis over oxidative phosphorylation.^21^

As a proxy for stoichiometry, we next quantified each protein’s abundance across BN-PAGE slices and overlaid these profiles with the apparent molecular masses of 20 major assemblies (Figure 1E; Table S4). Annotated subunits showed co-migration with their respective complexes, including the large and small mitoribosomal subunits (mt-LSU and mt-SSU), respiratory complexes, the RNA editing catalytic complex, the mitochondrial outer membrane translocase (ATOM), and the α-ketoacid dehydrogenase complex (KDH). Uniformly essential assemblies, such as ATOM, exhibited similar migration profiles across species, whereas shifts in the size and abundance of complex III (CIII) reflected species- and stage-specific differences in respiratory physiology.

In concert, these datasets provide a comparative atlas of mitochondrial complexomes from the evolutionarily divergent kinetoplastids *T. brucei* and *L. tarentolae*, including two developmental stages of *T. brucei* with distinct mitochondrial functions.

### Comparative complexome and structural analyses of mitochondrial assemblies

To assess the apparent masses, heterogeneity, and assembly trajectories of mitochondrial complexes, we compared complexome profiles of representative ribonucleoprotein assemblies, including the mitoribosomal subunits and the RNA editing substrate-binding complexes, and the multisubunit 3-methylcrotonyl-CoA carboxylase (MCC) protein complexes, which exhibit distinct oligomerization properties in *T. brucei* and *L. tarentolae*, with their published cryo-EM structures.

The kinetoplastid mitoribosome is one of the largest molecular machines, with an estimated mass of ~4.5 MDa. Cryo-EM identified 127 mitoribosomal proteins (mt-RPs) and 66 assembly factors (AFs) in TbPCF^3,22^ and 126 mt-RPs and 63 AFs in LtPMF.^6^ Consistent with these structures, our complexome profiling detected 123 mt-RPs in TbPCF, 115 in TbBSF, and 123 in LtPMF, together with 62, 54, and 61 corresponding AFs, respectively (Figures 2A and 2B; Table S2). As predicted from cryo-EM reconstructions, the mature mt-SSU migrated at a lower mass (~1.2 MDa) than the mt-LSU (~1.4 MDa), while mt-SSU assembly intermediates containing respective AFs appeared at larger masses (~1.7 MDa) than mt-LSU intermediates (~1.5 MDa). These patterns recapitulate the structural and temporal asymmetry of mitoribosome biogenesis: the RNA-rich mt-SSU assembles earlier through multiple extended intermediates, whereas the mt-LSU proceeds through a more compact late-stage maturation pathway.^23^

In TbPCF, mt-SSU biogenesis includes early assemblosome, intermediate, and late maturation states, which cryo-EM has resolved.^6,22–24^ Consistent with these structures, complexome profiling in LtPMF and TbPCF revealed dynamic association of mt-SSU assembly factors (mt-SAFs), including SAF4, SAF9, SAF12, SAF15, SAF16, SAF17, SAF19, SAF20, SAF21, SAF32, SAF34, SAF36, and mt-IF2, with the high–molecular mass assemblosome, whereas most mt-SSU ribosomal proteins remained embedded within the mature subunits (Figure 2A; Table S2). Except for SAF36 and mt-IF2, these SAFs are solvent-exposed, and their dissociation likely reflects the transition from the early assemblosome to intermediate and late stages, which are characterized by recruitment of additional mt-RPs and late-acting AFs. SAF36 and mt-IF2 persist into later intermediates, in agreement with their roles in mt-SSU maturation.^25^

Similarly, cryo-EM has visualized precursor states “A” and “B” during mt-LSU assembly. Complexome profiles revealed progressive dissociation of mt-LSU assembly factors (mt-LAFs) in both TbPCF and LtPMF (Figure 2B). In TbPCF, LAF23 and LAF24 (state A), LAF9 (state B), and mt-ACP, LAF8, LAF12, and LAF18 (state A/B) dissociated as expected. In LtPMF, LAF23 and LAF24 (state A), together with mt-ACP, LAF8, LAF15, and LAF18 (state A/B), as well as LAF9 (state B), displayed analogous behavior. The orderly loss of these factors from co-migration peaks reflects the sequential remodeling events underlying mt-LSU maturation.

Complexome profiling also resolved RESCs defined by cryo-EM in TbPCF: the gRNA-stabilizing RESC-A complex and the gRNA–mRNA annealing RESC-B complex (Figure 2C; Table S2).^7^ The gRNA-binding RESC1/2 heterodimer^26^ displayed a discrete peak at ~0.18 MDa in all three samples, consistent with its structure.^27^ However, most RESC-A (RESC1–6) and RESC-B (RESC5–14) subunits did not co-migrate as distinct particles of ~400 kDa and ~600 kDa, as predicted from cryo-EM structures.^7^ Instead, all RESC-A and RESC-B components, except RESC7, predominantly clustered at ~1.2 MDa. This larger assembly identifies a RESC-A/B supercomplex containing components of both RESC-A and RESC-B, suggesting functional coupling between gRNA stabilization and mRNA editing.

To further illustrate the utility of complexome profiling, we examined the polyadenylation mediator complex (PAMC), which includes RESC15–18 and was predicted by affinity purification–mass spectrometry to interact with RESC.^28^ In BN-PAGE, RESC15–18 co-migrated in a pattern concordant with both the formation of a discrete ~400-kDa PAMC module and interaction of this module with the RESC in *T. brucei;* this association was less apparent in *L. tarentolae*.

We next analyzed enzymes known to undergo metabolism-dependent oligomerization. Comparison of complexome profiles with cryo-EM structures revealed concordant species-specific organization of the leucine-catabolizing MCC (Figure 2D). The ~80 kDa MCC-α subunit migrated at ~1.1 MDa in LtPMF, whereas in TbPCF and TbBSF it displayed an apparent mass of ~0.6 MDa. MCC-β also showed a distinct migration pattern, appearing as a sharp band within a slightly larger particle in LtPMF (~0.6 MDa), whereas in TbPCF and TbBSF it predominantly migrated at ~0.4 MDa. These profiles recapitulate cryo-EM–defined MCC architectures, including catalytically inactive elongated filaments in LtPMF^10^ and the active α6β6 oligomer in TbPCF.^9^ In addition, the α-ketoacid dehydrogenase (KDH) complex migrated at ~1.6 MDa, reflecting oligomerization of the E2 core with associated E1/E3 modules, closely paralleling the organization of its mammalian counterpart^29^ (Table S2).

Overall, these analyses demonstrate that complexome profiling not only accurately captures the composition of mitochondrial assemblies visualized by cryo-EM but also delineates species-specific differences and higher-order interactions that may not be readily amenable to direct structural determination.

### Prediction of species-specific complex components by complexome profiling

Most of the 66 annotated CI subunits were detected by complexome profiling (43 in LtPMF, 56 in TbPCF, and 47 in TbBSF), yet none of the samples showed a complex at the expected >1-MDa mass of the CI holoenzyme. Instead, CI subunits were distributed across a broad mass range, consistent with the fragmented Complex I architecture reported in trypanosomatids.^30–32^

Respiratory complexes CII, CIII, and CIV displayed discrete co-migration profiles in both LtPMF and TbPCF, with peaks at ~400, ~504, and ~565 kDa, respectively (Figure 3A; Table S2). As expected, TbBSF lacked the cytochrome-containing complexes III and IV because of impaired editing of the corresponding mitochondrial mRNAs and its reliance on glycolysis, whereas both complexes were detected in the actively respiring LtPMF and TbPCF. Complexes II and V were observed across organisms, with all 14 annotated CII subunits and 25–26 of the 31 CV subunits identified.

We next leveraged our datasets to predict species-specific subunits of otherwise conserved respiratory complexes. For complexes II–V, we generated “bait” co-migration profiles by calculating the relative abundance of all annotated subunits across BN-PAGE slices. The iBAQ values were normalized to the maximal signal for each protein and averaged across the corresponding bait subunits. All remaining proteins in the dataset were then scored for similarity to the bait profile using Pearson correlation. Proteins with correlation coefficients >0.95 were considered candidate subunits and subjected to manual curation (Figure 3B).

This approach identified 19 candidate subunits of complexes II–V (Figure 3B; Table S2). Of these, 11 were further supported by co-migration in TbPCF: one of two complex II candidates (Tb927.9.8815), five of seven complex III candidates (Tb927.4.4990, Tb927.3.700, Tb927.10.4240, Tb927.11.16510, and Tb927.5.2560), and five of nine complex IV candidates (Tb927.10.6200, Tb927.7.7090, Tb927.11.2930, Tb927.11.13125, and Tb927.10.13230).

Among the 19 candidates, several displayed expression patterns characteristic of authentic oxidative phosphorylation (OXPHOS) subunits. The CIV candidate Tb927.10.6200 co-expressed with other CIV genes,^33^ whereas the CIV candidates Tb927.10.6200 and Tb927.7.7090, together with the CIII candidates Tb927.10.4240, Tb927.5.2560, and Tb927.11.16510, were co-depleted following knockdown of the mitoribosome receptor Mba1.^34^ These concordant expression and abundance patterns also support their incorporation into the OXPHOS system.

In this analysis, we also noted that the 62-aa CV subunit ATPEG4 in *T. brucei*,^12^ lacked an annotated ortholog in *L. tarentolae*. Suspecting an annotation gap, we re-examined the *L. tarentolae* genome and identified an open reading frame (LtaP27:632926–633111) with predicted sequence similarity to ATPEG4 and whose protein product co-migrated with CV in LtPMF, supporting its identity as the missing ATPEG4 ortholog. More broadly, co-migration of species-specific candidates with established subunits fortifies their assignment to the corresponding respiratory complexes.

### Structures of CIII_2_, CIV_2_, and CV reveal conserved and lineage-specific subunits

Complexome profiling across kinetoplastid models indicated that respiratory complexes are especially abundant and homogeneous in LtPMF, including both kDNA- and nuclear-encoded proteins. To define the architecture of these assemblies, we performed single-particle cryo-EM on unfractionated mitochondrial lysate from *L. tarentolae*. Without targeted purification, we obtained high-resolution reconstructions of Complex III_2_ (CIII_2_), Complex IV_2_ (CIV_2_), and ATP synthase (CV) from a single sample (Figure 4; Table S5).

Particles corresponding to CIII_2_ and CIV_2_ were refined to global resolutions of 3.0 Å and 3.2 Å, respectively. For CV, additional masked refinements focused on the dimer membrane region, the F_1_/central stalk, the F_o_ monomer membrane region, and the peripheral stalk, yielding local resolutions of 3.6–4.8 Å. Focused classification of the F_1_/central stalk identified two distinct rotational states refined to 4.2 Å and 4.1 Å, while the refinement of the dimer membrane region reached 3.7 Å and enabled detailed modeling of the dimer interface (Figure S1; Table S5).

The cryo-EM maps supported confident model building for 11 subunits in CIII_2_, 17 in CIV_2_, and 15 in CV F_*o*_ membrane region using ModelAngelo.^35^ The density quality enabled *de novo* sequence assignment, confirming complexome-derived predictions: five candidates in CIII_2_ (LtaP31.3010, LtaP27.0110, LtaP35.0250, LtaP32.3800, and LtaP17.1410), and three in CIV_2_ (LtaP36.1760, LtaP05.0800, and LtaP33.2260) (Figures 3B, S2D, S2E, S2G, and S2H; Tables S6 and S7). Additional kinetoplastid-specific subunits were identified across all three complexes, underscoring lineage-specific remodeling of the respiratory machinery (Figures 5A, 6A, and 7A).

Five kinetoplast DNA-encoded subunits were resolved in our structures (Figures S2A–S2C). In CIII_2_, cytochrome b (CYB), translated from a moderately edited mRNA, forms the transmembrane catalytic core that contains the Q_i_ sites. In CIV_2_, COX1, COX2, and COX3, originating from unedited, moderately edited, and pan-edited transcripts, respectively, were visualized. COX1 harbors the CuB catalytic center for O_2_ reduction; COX2 binds cytochrome c through its CuA center on the intermembrane-space side; and COX3 contributes to the transmembrane proton translocation. In CV, the ATP6 subunit encoded by a pan-edited mRNA interacts with the c-ring and the peripheral stalk, forming the structural linkage that couples proton translocation to rotary ATP synthesis.

### CIII_2_ exhibits a conserved catalytic core with lineage-specific elaborations

The cryo-EM structure of LtPMF CIII_2_ reveals a conserved catalytic core characteristic of eukaryotic cytochrome b complexes, in which each monomer contains two heme groups and one quinone-binding site, while also displaying kinetoplastid-specific adaptations. The complete model comprises 11 subunits, four of which are restricted to kinetoplastids (Figure 5A; Table S6).

The mitochondrial processing peptidase (MPP) heterodimer within CIII_2_ lacks the canonical substrate-binding pocket and catalytic motifs that define active peptidases^36–40^ (Figure S3). Instead, its substrate-binding cavity is occupied by the clade-specific protein QCR8 (LtaP35.0250), whose N-terminal extension (residues 13–28) inserts deeply into the MPP heterodimer (Figures 5B–5D and S3A–S3C).^37–40^ This architecture mirrors the structural, rather than enzymatic, role of MPP pseudoenzymes in *S. cerevisiae* and humans, yet contrasts sharply with catalytically active MPPs in *T. gondii* and *A. thaliana* (Figure S3D–S3E).^36–39^ These comparisons suggest that kinetoplastid and fungal MPPs have independently diverged from their ancestral protease function to assume a structural role within the CIII_2_ dimer.

The Q_i_ site of cytochrome b has been identified as the target of the antileishmanial compound DNDI-6174, which is also active against *T. cruzi*.^41^ To investigate the structural basis of CIII_2_ inhibition and the mechanism of resistance to this compound, we performed molecular docking of DNDI-6174 into the LtPMF CIII_2_ Q_i_ site. In our docking model, the inhibitor’s aromatic ring aligns parallel to Phe223, enabling a π–π stacking interaction that anchors the compound, while Phe34, Tyr30, Leu201, and Leu198 contribute additional hydrophobic contacts that stabilize the ligand within the Q_i_ cavity (Figure S3G). Resistance-associated mutations^42^ cluster along one face of the Q_i_ pocket (Figure S3H), highlighting a region critical for inhibitor engagement. Although Q_i_-site inhibitors have also been characterized in apicomplexan CIII_2_ complexes,^38^ conservation of the DNDI-6174 binding pocket between LtPMF and *T. brucei*, together with its divergence from mammalian and apicomplexan counterparts, provides a rationale for kinetoplastid selectivity.

### CIV_2_ adopts a conserved catalytic core with a lineage-specific dimerization interface

The LtPMF CIV_2_ exhibits a distinct overall organization compared with homologous complexes in other eukaryotes, including an enlarged monomer with pronounced matrix-facing extensions and multiple kinetoplastid-specific subunits that remodel the dimer interface (Figures 6A–6C and S4).^38,43–45^ Despite these innovations, the catalytic core of CIV, comprising the kDNA-encoded subunits COX1, COX2, and COX3, retains an overall structural arrangement conserved from yeast to mammals (Figure S4C).^38,43,44^ The complete LtPMF model contains 17 subunits, of which 13 are kinetoplastid-specific (Figure 6A; Table S7).

Dimerization in LtPMF CIV_2_ is mediated primarily by a network of aromatic and electrostatic interactions involving P27, COX3, COX6, COX9, and LtaP33.2260 (Figures 6B–6D). Trp143 of P27 in monomer 2 engages in π-stacking with Trp143 of P27 and Phe277 of COX3 in monomer 1, while the reciprocal Trp143 from monomer 1 stacks against Tyr286 of COX3 in monomer 2 (Figure 6D). In addition, Phe135 on an extra β-sheet of COX3 forms symmetric Phe135–Phe135 interactions across the interface (Figures 6D, S4A and S4B).^44,45^ Electrostatic contacts between Glu104 and Lys98 of COX6 (Monomer 1) and Arg114 and Asp117 of COX9 (monomer 2) further reinforce the interface (Figure 6D). Together, these π-stacking and charge-pairing interactions generate a robust dimerization interface not observed in other organisms (Figure S4C).^38,43,44^

LtPMF COX1 also contains a C-terminal extension absent from other eukaryotic homologs, which may help stabilize its unusually large monomeric unit (Figures 6E–6G).^38,44,45^ Together, these clade-restricted structural features indicate that kinetoplastid CIV_2_ evolved a specialized dimeric architecture distinct from that of opisthokont and plant lineages.^38,43–45^

In mammals, the monomer–dimer equilibrium has been linked to reversible phosphorylation, with phosphorylation sites identified in COX1, COX2, COXIV, COXVa, and COXVb.^46–51^ Several of these sites are conserved in LtPMF CIV, including Tyr306 in LtCOX1 (corresponding to Tyr304 in bovine COX1) and Ser122 in LtCOX2 (Ser126 in bovine COX2).^52,53^ Because phosphorylation-associated regulation of mammalian CIV is not restricted to residues located directly at the dimer interface, the absence of these conserved sites from the *L. tarentolae* CIV_2_ interface does not preclude indirect or allosteric effects on dimerization.

### CV forms a compact dimer stabilized by a remodeled peripheral stalk

The LtPMF ATP synthase (CV) forms a dimer with an inter-monomer angle of ~60° between the two F_1_/C_10_-ring subcomplexes (Figure 7B). This geometry differs from the wide-angle dimers characteristic of opisthokonts but closely resembles that of the TbPCF ATP synthase.^12,54^ The overall LtPMF CV architecture shown in Figures 4C and 7A includes the F_1_/central-stalk, F_0_ membrane, and peripheral-stalk regions. The F_1_/central-stalk is represented by a model fitted into the corresponding density without subsequent map-guided rebuilding or refinement. The LtPMF CV F_*0*_–peripheral-stalk model contains 18 subunits, 15 of which are kinetoplastid-specific (Figure 7A; Table S8).

The F_o_ region features a C_10_ ring coupled to the mitochondrially encoded ATP6 subunit (Figure 7C). At the proton-translocation interface, the conserved electrostatic pair Arg146 (ATP6) and Glu60 (c-subunit) mediates proton handoff between half-channels, functionally equivalent to the canonical aArg–cGlu interaction (e.g., aArg176–cGlu59 in *S. cerevisiae*).^54^ In *T. brucei*, the corresponding arginine in the ATP6 subunit contacts cGlu102, underscoring conservation of the proton-handover mechanism across kinetoplastids.^12^

A major point of divergence lies in the organization of the peripheral stalk and transmembrane dimer interface. The long bH2 helix that forms the core of the peripheral stalk in most eukaryotes is absent in both LtPMF and TbPCF. Instead, the kinetoplastid-specific subunit ATPEG4 occupies this structural position (Figures 7D, S2F and S2I).^12,54^ In LtPMF, the canonical subunit b consists of only a single transmembrane helix (bH1), which anchors subunits e and g. Replacement of the bH2 helix by ATPEG4 results in a peripheral stalk architecture and dimer interface distinct from that of opisthokonts^54^ and Acanthamoeba.^55^

Electrostatic surface comparisons reveal pronounced charge differences between the e/g modules of LtPMF and TbPCF (Figure 7E). The LtPMF g-subunit exhibits a more positively charged surface than its TbPCF counterpart.^12^ In *T. brucei*, subunit g is required for dimer stability and cristae morphology: RNAi-mediated knockdown disrupts ATP synthase oligomerization and produces aberrant, rounded cristae, phenotypes analogous to those observed upon loss of subunits e or g in *S. cerevisiae*.^12,56^ The spatial arrangement of the e/g subunits is nearly identical in LtPMF and TbPCF, with both pairs positioned symmetrically along the dimer axis, contrasting with the laterally displaced e/g modules in yeast and mammals.^54,57–59^

In kinetoplastids, conserved GXXXG motifs within the transmembrane helices of subunits e and g position these helices in close apposition, directly contributing to dimer stabilization.^12^ The LtPMF ATP synthase dimer adopts a compact membrane-domain interface geometry similar to that reported for TbPCF.^12^ Together with the higher-order assemblies observed by complexome profiling, this conserved architecture suggests that kinetoplastid ATP synthases may also assemble into higher-order oligomers (Figure 3), which may contribute to the sharply curved cristae rims characteristic of the kinetoplastid mitochondrion.^12,60^

## DISCUSSION

This study provides a systems-level characterization of mitochondrial macromolecular assemblies in the model kinetoplastids *T. brucei* and *L. tarentolae*. By integrating recently established MitoCarta inventories^5^ with complexome profiling (BN-PAGE-MS) and single-particle cryo-EM, we define how nuclear- and kDNA-encoded proteins assemble into macromolecular complexes across species and developmental stages. Our approach refines and expands existing annotations, resolves the structural and compositional heterogeneity of mitochondrial assemblies, and uncovers lineage-specific innovations and previously unrecognized interactions that shape mitochondrial architecture and function in Kinetoplastea.

In kinetoplastids, the mitochondrial proteome comprises numerous protein and ribonucleoprotein assemblies; however, cryo-EM structures have been determined for only a subset, including the mitoribosome,^3,6,22,61^ the RESC^7^ and RECC^8^ RNA editing complexes, 3-methylcrotonyl^9,10^- and propionyl-CoA carboxylases^11^ and the *T. brucei* ATP synthase.^12^ Among these, only the mitoribosome and MCC have been structurally resolved in both *Trypanosoma* and *Leishmania*, genera that diverged approximately 120 million years ago. Here, we use complexome profiling to assess the structural diversity, assembly intermediates, and interactions of 20 established mitochondrial complexes and to uncover previously unrecognized, lineage-specific components of conserved molecular machines.

The co-migration profiles of mitoribosomal complexes revealed mature subunits and associated assembly intermediates, a pattern consistent with the asymmetric biogenesis trajectory of the kinetoplastid ribosome.^61^ The kinetoplastid proteome includes more than 50 members of the pentatricopeptide repeat (PPR)^62^ protein family (Table S2), which is massively expanded in land plants.^63,64^ Fifteen PPR proteins have been visualized as ribosomal proteins or assembly factors in cryo-EM reconstructions of the TbPCF mitoribosome.^3,22,24^ Complexome profiling across *T. brucei* and *L. tarentolae* suggests that at least two previously unannotated PPR proteins co-migrate with the mt-SSU assemblosome and likely function as assembly factors. In addition, BN-PAGE-MS detected co-migration of the mRNA poly(A)-binding protein KPAF4,^14^ a 32-kDa PPR protein, at an apparent molecular mass exceeding 2.5 MDa in TbPCF, possibly indicating its association with the ribosome.

The RNA editing substrate-binding complex profiles matched those of the RESC1/2 heterodimer,^27^ the six-subunit gRNA-binding RESC-A complex, and the 12-subunit gRNA–mRNA annealing RESC-B complex.^7^ However, co-migration of most RESC proteins resolved in these TbPCF cryo-EM structures revealed a predominant ~1.2 MDa RESC-A/B superassembly containing components of both RESC-A and RESC-B (Table S2). Likewise, the higher-than-predicted apparent mass of the KDH complex (~1.6 MDa) suggests multienzyme oligomerization, consistent with prior reports.^29^ In contrast, the RNA editing catalytic complexes exhibited defined co-migration profiles in all three datasets, indicating that the ~1.1 MDa RECCs^8,65^ maintain a highly conserved architecture across species.

BN-PAGE-MS confirmed the heterogeneous nature of complex I across all three organisms, consistent with reports of incomplete assemblies in kinetoplastids.^32^ In contrast, respiratory complexes CII–CV displayed narrow size distributions. Based on co-migration profiles, we nominated two previously unassigned proteins for CII, seven for CIII, nine for CIV, and one for CV in LtPMF (Figure 3B). Among these, five CIII_2_ candidates, three CIV_2_ candidates, and one CV candidate were visualized in cryo-EM structures (Figures 4 and S2D–S2I).

The LtPMF respiratory complex structures show varied strategies by which lineage-restricted elaborations augment conserved proton-translocating cores. In CIII_2_, the N-terminal extension of QCR8 inserts into the inactive MPP*α*/*β* heterodimer, reinforcing dimer stability (Figure 5B–5D). The loss of proteolytic activity in MPP*α*/*β*, which now functions exclusively as a structural element, parallels the evolutionary trajectory observed in fungi, but contrasts with catalytically active MPPs in plants and apicomplexans^36–39,43,66^ (Figure S3D–S3F). The extensive CIV_2_ dimer interface visualized by cryo-EM suggests that dimerization is an integral architectural feature of kinetoplastid CIV (Figures 3B and 6). This dimeric state may modulate electron flux through CIV and thereby regulate the respiratory output. Alternatively, the CIV_2_ architecture may play a role analogous to that of the transient CIII–CIV supercomplexes observed in other eukaryotes.^67^ This interpretation is consistent with the lineage-specific structural elements that reinforce the dimer interface in kinetoplastid CIV_2_.

By integrating complexome profiling with cryo-EM analysis of unfractionated mitochondrial lysates, this work establishes a broadly applicable framework for defining how conserved and lineage-restricted mitochondrial proteins assemble into functional complexes. This approach provides a foundation for mechanistic studies in kinetoplastids and for dissecting parasite-specific features with therapeutic potential.

## LIMITATIONS OF THE STUDY

We ran the BN-PAGE gels used for complexome profiling of LtPMF, TbPCF, and TbBSF independently, although each was calibrated against the same protein standards. Consequently, subtle discrepancies in apparent molecular mass assignments across datasets cannot be excluded. To preserve the architecture of respiratory complexes in LtPMF, we solubilized freshly isolated mitochondria immediately before BN-PAGE separation or cryo-EM grid preparation; however, detergent extraction may still disrupt higher-order assemblies or destabilize low-affinity interactions. The absence of additional biochemical enrichment steps may also have limited the detection of small or low-abundance complexes by both complexome profiling and cryo-EM. Finally, we performed structural analyses in LtPMF, and the extent to which these architectural features vary across other kinetoplastid species or life-cycle stages remains to be established.

## RESOURCE AVAILABILITY

### Lead contact

Requests for further information should be directed to the lead contact, Ruslan Aphasizhev (ruslana@bu.edu).

### Materials availability

This study did not generate new unique reagents.

### Data and code availability

- The cryo-EM maps and corresponding atomic models have been deposited at Electron Microscopy Data Bank (EMDB) and Protein Data Bank (PDB), respectively: *L. tarentolae* respiratory complex III_2_ (EMD-73948; PDB: 9Z9K), respiratory complex IV_2_ (EMD-73966; PDB: 9ZAK), ATP synthase F_*o*_ dimer membrane region (EMD-73974; PDB: 9ZB4), ATP synthase peripheral stalk (EMD-74030; PDB: 9ZC4), ATP synthase F_*o*_ monomer membrane region (EMD-74031; PDB: 9ZC5), ATP synthase F_1_/central-stalk cryo-EM density (EMD-78449), and ATP synthase dimer cryo-EM density (EMD-78468). Mass spectra and protein sequence databases used for the searches have been deposited in MassIVE repository under accession number MSV000102789. They are publicly available as of the date of publication. Blue native PAGE images have been deposited at the Mendeley Data repository at https://doi.org/10.17632/d5ywdrwzkn.2 and are publicly available as of the date of publication.
- This paper does not report original code.
- Any additional information required to reanalyze the data reported in this paper is available from the lead contact upon request.

## STAR METHODS

### EXPERIMENTAL MODEL AND STUDY PARTICIPANT DETAILS

*Leishmania tarentolae* promastigote (LtPMF), *Trypanosoma brucei brucei* Lister 427 procyclic (TbPCF), and bloodstream (TbBSF) forms were used in this study. LtPMF were grown in brain heart infusion (BHI) broth supplemented with 10 μg/ml hemin at 27 °C and collected at a density of ~5.5 × 10^7^ cells/ml. TbPCF parasites were cultured in SDM-79 medium supplemented with 10% fetal calf serum (FCS) at 27 °C and harvested at a cell density of ~3 × 10^7^ cells/ml. The TbBSF was maintained in HMI-9 medium containing 15% FCS at 37 °C, and collected at ~2.4 × 10^6^ cells/ml.

### METHOD DETAILS

#### Isolation of mitochondria

Cells were harvested at logarithmic growth phase by centrifugation (3,000 × g, 15 min, 4 °C) and washed once with cold PBS. Pellets were resuspended in SoTE buffer (0.6 M sorbitol, 20 mM Tris-HCl, 2 mM EDTA) and lysed in a nitrogen cavitation vessel (90 bar for LtPMF and 85 bar for TbPCF and TbBSF, 30 min). Lysates were treated with DNase I (40 U/ml, 20 min, on ice) and benzonase (5U/ml), and the reaction was terminated by adding EDTA (6 mM final concentration). The lysates were centrifuged at 15,000 × g for 15 min at 4 °C, and the pellet was resuspended in SoTE. This suspension was centrifuged at low speed (560 × g, 20 min, 4 °C, swing-bucket rotor) to remove unbroken cells and debris. The supernatant was transferred to fresh tubes and centrifuged at 15,000 × g for 15 min at 4 °C to obtain crude mitochondrial pellets. For further purification, crude pellets were resuspended in SoTE and subjected to continuous density gradient centrifugation (96,600 × g, 30 min, 4 °C, SW41Ti rotor) using Optiprep (iodixanol) gradients: 10–30% for LtPMF and 5–20% for TbPCF and TbBSF. Mitochondrial bands were collected by side puncture with an 18G needle, washed with SoTE, snap-frozen in liquid nitrogen, and stored at −80 °C until use for BN-PAGE.

#### Blue Native (BN)-PAGE

Mitochondrial pellets were solubilized at 10 μg/μl in lysis buffer (0.6 M sorbitol, 20 mM Tris–HCl pH 7.5, 10 mM MgCl_2_, 0.5 mM CaCl_2_, 1 U/ml Turbo DNase) supplemented with 0.25 mM PMSF (100 mM stock in ethanol) and 1X EDTA-free protease inhibitor cocktail (50X stock, 1 tablet in 1 mL H_2_O). Protein concentrations of the mitochondrial pellet were determined using the Pierce^™^ BCA Protein Assay Kit (Thermo Fisher Scientific, Cat. No. PI23225) according to the manufacturer’s instructions. For electrophoresis, 40 μl of sample was prepared at a detergent/protein ratio of 2 (g/g) by mixing 10 μl of lysed mitochondria, 10 μl of NativePAGE 4× Sample Buffer, 2 μl of n-dodecyl-β-D-maltoside (DDM), and 18 μl of H_2_O. Samples were incubated on ice (20 min, 4 °C) and centrifuged (20,000 g, 10 min, 4 °C). The resulting supernatant (35 μl) was transferred to a fresh tube on ice, and 1 μl of NativePAGE 5% G-250 Sample Additive was added.

Electrophoresis was performed using the XCell SureLock Mini-Cell system in a cold room (4 °C). NativePAGE Novex 3–12% Bis-Tris protein gels were assembled in the chamber. A small stir bar was placed in the outer buffer compartment. Dark Blue Cathode Buffer (prepared from 10 ml 20× NativePAGE Running Buffer, 10 ml 20× NativePAGE Cathode Additive, and 180 ml deionized water, chilled to 4 °C) was used to rinse the gel wells (1 ml) and to fill the inner chamber (20 ml) for leak inspection. Samples were loaded as follows: LtPMF (20 μg protein), TbPCF (15 μg protein), and TbBSF (40 μg protein). The inner chamber was filled with Dark Blue Cathode Buffer, and the outer chamber was filled with Anode Buffer (25 ml 20× NativePAGE Running Buffer diluted to 500 ml with deionized water, 4 °C).

The gel was initially run at 150 V for 30 min (4 °C, with continuous stirring of the anode buffer). The run was paused, the Dark Blue Cathode Buffer was replaced with Light Blue Cathode Buffer (10 ml 20× NativePAGE Running Buffer, 1 ml 20× NativePAGE Cathode Additive, 189 ml deionized water, 4 °C), and electrophoresis was resumed at 250 V for 180 min (4 °C, with stirring).

Following electrophoresis, the gel was fixed in Fixation Solution (40% [v/v] methanol, 10% [v/v] acetic acid, 10 mM ammonium acetate) for 30 min at room temperature with gentle rocking. The gel was then destained in 10% (v/v) acetic acid (3 × 20 min, room temperature, gentle rocking) until the background was transparent and the NativeMark standard was visible. The gel was washed in deionized water (3 × 5 min, room temperature, gentle rocking) and imaged.

For subsequent processing, a cutting template of 60 slices was digitally prepared and printed. A 96-well filter microtiter plate was preconditioned with 100 μl of 100% acetonitrile and centrifuged (1,500 g, room temperature). The plate was washed three times with 100 μl of 50 mM ammonium bicarbonate (LC-MS grade water) and centrifuged (1,500 g, room temperature). Wells were then filled with 150 μl of 50 mM ammonium bicarbonate, covered, and placed on ice.

The gel was placed on a cutting board aligned with the template. Lanes of interest were excised using a sterile razor blade and divided into five sections. Beginning with the fragile upper section, individual gel fragments were cut horizontally with a scalpel. Each band was further cut into 2–3 cubes and transferred into the 96-well plate. Tools were washed with LC-MS-grade methanol and water between cuts to prevent contamination. The 96-well plate was stored at −80 °C.

#### BN-PAGE-MS gel slice processing and LC-MS/MS analysis

Gel bands were destained by incubating with 100 μl of 50% acetonitrile (ACN) at 400 rpm for 15 min at room temperature, followed by washing with 100 μl of 50 mM ammonium bicarbonate (AB) under identical conditions. This destaining-washing cycle was repeated three times until the gels became fully transparent. For protein reduction and alkylation, bands were treated with 50 μl of 10 mM dithiothreitol (DTT) at 37 °C for 45 min with shaking, then alkylated with 50 μl of 55 mM iodoacetamide in the dark for 1 h at room temperature. Samples were subsequently washed with 50% ACN and AB as described above and dehydrated in 100 μl of 100% ACN for 10 min at room temperature. After removing the solvent, gel pieces were rehydrated in 10 μl of trypsin solution (5 ng/μl) and incubated at 37 °C for 5 min, followed by addition of 50 μl AB to cover the gels. Proteolytic digestion proceeded overnight at 37 °C with shaking at 600 rpm.

The next day, supernatants were transferred to micronic vials. Remaining peptides were extracted sequentially from the gel pieces using 50 μl HPLC-grade water, 50 μl of 30% ACN/0.1% formic acid (FA), and 50 μl of 70% ACN/0.1% FA, each for 20 min at room temperature and 400 rpm. All extracts were pooled, frozen, and lyophilized for LC-MS/MS analysis.

Dried peptides were reconstituted in 45 μl of 0.1% FA, and 20 μl was loaded onto Evotips following the manufacturer’s protocol. LC-MS/MS analysis was performed on an Evosep One system coupled to a Q Exactive^™^ Plus Hybrid Quadrupole-Orbitrap^™^ Mass Spectrometer. Peptide separation was achieved on an Endurance column (15 cm × 0.15 mm, 1.9 μm) using the standard 30SPD method.

Data were acquired in data-dependent mode with an MS1 scan range of 300–1800 m/z at a resolution of 70,000. The automatic gain control (AGC) target was set to 3 × 10^6^ and the maximum injection time to 50 ms. The top 12 most intense ions per MS1 scan were selected for fragmentation with a normalized collision energy of 25 and an isolation window of 1.7 m/z. MS2 spectra were collected in centroid mode at 17,500 resolution, using an AGC target of 5 × 10^4^ and a 100 ms maximum injection time. Dynamic exclusion was set to 10 s, and ions with unassigned, singly charged, or >6 charges were excluded.

#### BN-PAGE-MS data processing

Mass spectrometry data were analyzed using the Spectrum Mill software (Broad Institute). Raw spectra were extracted within a precursor mass range of 600–6000 Da, retaining signals with a minimum MS1 signal-to-noise ratio of 25. MS1 spectra were merged when their retention times differed by less than ±45 s or precursor m/z values by less than ±1.4 m/z. Database searches were performed against *Leishmania tarentolae* (TriTrypDB-68_LtarentolaeParrotTarII_AnnotatedProteins.fasta*) and Trypanosoma brucei* TREU927 (TriTrypDB-68_TbruceiTREU927_AnnotatedProteins.fasta, limited to the longest protein isoform per gene locus). Enzyme specificity was set to “trypsin allow P” and up to four missed cleavages permitted. Carbamidomethylation of cysteine residues was specified as a fixed modification, while variable modifications included N-terminal acetylation, methionine oxidation, pyro-glutamate formation from glutamine, deamidation of asparagine, and conversion of carbamidomethylated cysteines to pyrocarbamidomethyl derivatives. Both precursor and fragment mass tolerances were set at 20 ppm. Peptide-spectrum matches were filtered using a 1% false discovery rate (FDR) cutoff to ensure identification confidence.

#### BN-PAGE-MS data analysis

iBAQ values were calculated for all proteins. Slice 1 was uninformative and discarded from future analysis. Protein groups were expanded such that all proteins in the protein group receiving the same top score (thus indistinguishable peptide evidence) were considered as detected. Proteins not in LtMitoCarta or TbMitoCarta were excluded from analysis, as were 52 TbMitoCarta proteins located on suspicious chromosome Tb927_11_bin_v5.1 that are likely genome assembly artifacts^68,69^ (these had high identity sequence matches to proteins on properly assembled chromosomes). Relative abundance was calculated for each protein as iBAQ value / Max iBAQ value observed across any slice 2–60. For display purposes, orthologs across species were defined based on BLASTP^70^ best bidirectional hit, Expect < 1. Figures 1D–E were generated in R. For each experiment (LtPMF, TbPCF, and TbBSF), for each respiratory complex, we (i) identified marker proteins that showed a coherent profile in that experiment, (ii) created a “bait” profile as the average of these marker proteins in that experiment, (iii) scored all other proteins using Pearson correlation to the “bait” to that experiment, (iv) manually reviewed high-scoring proteins as potential co-migration hits.

#### Curation of known mitochondrial protein complexes

Mitochondrial protein complexes were curated by literature review. Searches were performed in PubMed and Google Scholar using the names of mitochondrial protein complexes and related keywords. Review articles were used to identify complexes and their subunits, and primary publications were consulted to evaluate supporting evidence. Proteins were classified as bona fide subunits when supported by at least one of the following criteria: identification by mass spectrometry following immunoprecipitation or affinity purification of a validated bait protein, or visualization within a cryo-EM structure. The curated subunit list was used for all analyses.

#### Identification of Pentatricopeptide Repeat (PPR) proteins

PPR proteins were identified using a combination of literature curation and computational analyses. Known *T. brucei* PPRs were compiled from published sources, and additional candidates were identified among MitoCarta proteins detected in BN-PAGE-MS. These were filtered by excluding proteins lacking an *L. tarentolae* ortholog, proteins <20 kDa, and those detected in slices corresponding to <200 kDa. The remaining 290 proteins were analyzed by retrieving structural models from the AlphaFold Protein Structure Database,^71,72^ or by generating predictions with AlphaFold 3.^73^ Each structure was compared against the PDB100 database using Foldseek,^74^ and proteins with PPR-related matches at probability ≥0.95 were classified as PPR candidates. In parallel, all 290 proteins were examined with TPRpred,^75^ retaining proteins with P-value ≤0.01 and posterior probability >1%. Proteins supported by either structural or sequence-based evidence were designated as assigned PPRs.

#### Cryo-EM sample preparation and data collection

Cryo-EM sample preparation was performed using an adapted protocol used for *A. castellanii* mitochondria.^55^ Briefly, density-gradient isolated mitochondrial fraction (0.4 mg of protein) was extracted in 50 μl of 50 mM HEPES pH 7.9, 300 mM NaCl, 4 mM MgCl_2_, 20 μM CaCl_2_, 0.1 mM Dithiothreitol (DTT), and 0.5% n-dodecyl-β-D-maltoside (DDM) for 10 min on ice, followed by centrifugation at 14,000 g for 10 min. The supernatant was then filtered through a 0.1 μm Durapore^®^ PVDF filter. For cryo-EM grid preparation, 3.5 μl of the filtered sample was applied to glow-discharged holey carbon support grids (Quantifoil 300 mesh, Cu R1.2/1.3). Grids were blotted for 4 s at 4 °C and 100% humidity (blot force: 15) and plunge-frozen in liquid ethane using a Vitrobot Mark IV (Thermo Scientific). Cryo-EM data were collected using a Titan Krios transmission electron microscope (Thermo Scientific) operated at 300 kV, equipped with a Gatan K3 direct electron detector and a 20 eV slit-width energy filter. A total of 43,589 movies were recorded in counting mode using EPU software. Data were acquired at a nominal magnification of 165,000×, corresponding to a calibrated pixel size of 0.736 Å, with a defocus range of −0.6 to −1.8 μm. The dose rate was ~12 e^−^/pixel/s, with a total accumulated dose of 52 e^−^/Å^2^.

#### Cryo-EM data processing

All raw movies were subjected to beam-induced motion correction and patch-based contrast transfer function (CTF) estimation using cryoSPARC v4.7.0.^76^ For CIII_2_, an initial subset of particles was manually picked, followed by 2D classification. Selected 2D classes were used as templates for automated particle picking, yielding 358,415 particles. Particles were extracted in a box size of 432 pixels and Fourier-cropped to 72 pixels for iterative rounds of two-dimensional (2D) classification to remove junk particles. Following ab initio 3D reconstruction with C1 symmetry, 13,544 particles were refined with imposed C2 symmetry via homogeneous refinement and subsequent non-uniform refinement, resulting in a consensus map with a resolution of 3.0 Å. For CIV_2,_ particle picking was performed using the template-free blob picker in cryoSPARC v4.7.0.^76^ A total of 230,494 particles was picked and subjected to iterative rounds of 2D classification. The selected particles (a total of 31,885 particles) were then subjected to ab initio 3D reconstruction into five classes with C1 symmetry. One of the five clusters (a total of 5,392 particles) was refined using non-uniform refinement with C2 symmetry, yielding a map at 3.21 Å resolution.

For CV (ATP synthase), particle picking was performed using the template-free blob picker in cryoSPARC v4.7.0.^76^ A total of 239,709 particles was picked and subjected to iterative rounds of 2D classification, followed by ab initio 3D reconstruction into three classes with C1 symmetry. Particles from each reconstruction were further subjected to an additional 2D classification. A total of 8,556 particles were selected and used for a second round of ab initio 3D reconstruction. The class displaying the expected architecture of an ATP synthase dimer (a total of 4,649particles) was refined using non-uniform refinement with C2 symmetry, yielding a map at 3.8 Å resolution. A mask focusing on the ATP synthase transmembrane dimer region was generated in UCSF ChimeraX v1.6^77^ and used for focused refinement, improving the resolution to 3.7 Å. For the ATP synthase monomer, the ATP synthase dimer particles underwent the C2-symmetry expansion, and a mask focusing on the monomer region was generated using UCSF ChimeraX v1.6,^77^ which was used for particle subtraction to isolate the cryo-EM map density of the ATP synthase monomer. These subtracted particles were subjected to homogeneous reconstruction only to get an initial map, followed by centering on the mask of the ATP synthase monomer using volume align tools. A total of 9,298 particles were re-extracted to a box size of 480 pixels and then subjected to ab initio 3D reconstruction into two classes. A final stack consisting of 8,358 particles was utilized for a non-uniform refinement, resulting in a consensus map with a resolution of 3.8 Å. The map was subjected to local refinements of the F_1_/central stalk, F_o_ monomer, and peripheral stalk, yielding maps at resolutions of 4.8 Å, 3.6 Å, and 3.9 Å, respectively. A mask focusing on F_1_/central stalk was generated in UCSF ChimeraX v1.6^77^ and used for 3D classification into three classes. Two classes exhibiting similar density (a total of 4,690 particles) were pooled and subjected to local refinement, resulting in a map at a resolution of 4.2 Å. The remaining class underwent local refinement, yielding a map with a resolution of 4.1 Å. The composite map was generated by aligning the locally refined F_1_/central stalk Rot1, F_o_ monomer membrane region and peripheral stalk to the consensus map, then combining the three locally refined maps using the maximum values of each component (vop maximum) in UCSF ChimeraX v1.6.^77^ The number of classes identified in this study may reflect the limited number of particles, rather than the complete conformational space of the complex.

#### Model building and refinement

The atomic model of CIII_2_ was built primarily using ModelAngelo (version 1.0.14)^35^, except for the intermembrane-space region of the Rieske protein (residues 217–291). Subunits with sequences annotated from complexome profiling by homology to *T. brucei*,^12^ were built with the ModelAngelo build command. For unannotated densities, *de novo* models were generated using the ModelAngelo build_no_seq command and subsequently searched against the *Leishmania tarentolae* Parrot Tar II proteome^78^ (TriTrypDB; https://tritrypdb.org/tritrypdb/app/). The cryo-EM density corresponding to the intermembrane-space region of the Rieske protein (residues 217–291) was weak and therefore insufficient for tracing by ModelAngelo^35^. An AlphaFold 3^73^ prediction was therefore used as a template for this region and fitted into the cryo-EM density using UCSF ChimeraX v1.6.^77^ All models underwent manual refinement through a combination of real-space refinement in Phenix 1.21^79^ and adjustments in ISOLDE^80^ and Coot.^81^ All final models were validated using MolProbity.^82^ Structural visualizations and superimpositions were generated with UCSF ChimeraX v1.6.^77^ Interactions between subunits were analyzed using the Residue Interaction Network Generator (RING).^83^

The atomic model of CIV_2_ was built using ModelAngelo (version 1.014).^35^ Subunits with sequences annotated from complexome profiling by homology to *T. brucei*,^12^ were built with the ModelAngelo build command. For unannotated densities, *de novo* models were generated using the ModelAngelo build_no_seq command and subsequently searched against the *Leishmania tarentolae* Parrot Tar II proteome^78^ (TriTrypDB; https://tritrypdb.org/tritrypdb/app/). All models underwent manual refinement through a combination of real-space refinement in Phenix 1.21^79^ and adjustments in ISOLDE^80^ and Coot.^81^ All final models were validated using MolProbity.^82^ Structural visualizations and superimpositions were generated with UCSF ChimeraX v1.6.^77^ Interactions between subunits were analyzed using the Residue Interaction Network Generator (RING).^83^

The atomic models of CV (ATP synthase) F_*o*_ dimer/F_*o*_ monomer were built using ModelAngelo (version 1.014).^35^ Subunits with sequences annotated from complexome profiling by homology to *T. brucei*,^12^ were built with the ModelAngelo build command. These included subunits *i/j*, ATPTB1, ATPEG3, *f*, ATPTB11, *k*, ATPTB6, *g*, subunit 8 (residues 39–104), *b*, ATPTB12, *e*, subunit c-1, A6, and ATPEG4 (LtaP27_632926_633111). The peripheral-stalk subunits—*d*, ATPTB3, subunit 8, and ATPTB4 were annotated from complexome profiling based on homology to *T. brucei*.^12^ The *T. brucei* peripheral-stalk structure (PDB 8AP8) was used as a starting model for building the LtPMF peripheral stalk. AlphaFold 3^73^ predictions of the corresponding LtPMF peripheral stalk subunits were fitted into the LtPMF ATP synthase reconstructions obtained by masked refinement of the peripheral-stalk region using UCSF ChimeraX v1.6.^77^ All models underwent manual refinement through a combination of real-space refinement in Phenix 1.21^79^ and adjustments in ISOLDE^80^ and Coot.^81^ All final models were validated using MolProbity.^82^ Structural visualizations and superimpositions were generated with UCSF ChimeraX v1.6.^77^ Interactions between subunits were analyzed using the Residue Interaction Network Generator (RING).^83^

The F_1_/central-stalk subunits, P18 (LtaP15.320), γ (LtaP21.2100), δ (LtaP30.3640), α (LtaP33.0850), β (LtaP35.0410), and subunit O/oligomycin sensitivity-conferring protein (OSCP; LtaP36.7010) were annotated from complexome profiling based on homology to *T. brucei*, and their structures were predicted using AlphaFold 3^73^. Models corresponding to rotational states 1 and 2 (Rot1 and Rot2) were assembled using the *T. brucei* ATP synthase structures in rotational state 1a (PDB: 8APA) and rotational state 2a (PDB: 8APF), respectively, as starting models. The AlphaFold 3-predicted LtPMF subunit models were positioned according to these starting models and fitted into the corresponding LtPMF cryo-EM densities using UCSF ChimeraX v1.6.^77^ The fitted models were not subjected to map-guided rebuilding or real-space refinement.

#### Molecular docking simulations

Molecular docking simulations were performed to predict the interaction sites of DNDI-6174 (SMILES: N#CC1=CC=C(C2=C(C)NC3=NC(N)=NC(C)=C32)C4=C1OCO4) with LtPMF CYB using RosettaLigand (version 3.7.1).^84,85^ All procedures followed the standard RosettaLigand docking protocol. Briefly, the ligand was converted from SMILES format to a .mol2 file using the Corina (Corina classic version; https://demos.mn-am.com/corina.html). Hydrogens were added with Antechamber in AmberTools.^86^ A ligand parameter file (ligand.params) and Rosetta-compatible conformations were generated using the molfile_to_params.py script with default parameters. The predicted DNDI-6174–CYB complex (holo.pdb) was generated via the PatchDock server.^87^ Docking was performed with RosettaScripts using an XML protocol (dock.xml) and an options.txt file specifying the input structures and ligand parameters. A total of 2000 docked models were generated with the following command: $ROSETTA3/bin/rosetta_scripts. linuxgccrelease@options.txt -nstruct 2000. The Rosetta score file (score.sc) was processed with the provided script extract_scores.sh to extract docking scores and RMSD values into a CSV file. The results were sorted to identify the best-scoring complexes: sort -t, -nk3 score_vs_rmasd.csv | head -n 20. The top-scoring ~1% of models (20 out of 2000) were subjected to clustering analysis, and representative binding poses were selected for further interpretation. Models in which the ligand overlapped with the heme group were discarded. The ligand–interaction diagram for the predicted binding mode of DNDI-6174 in the LtPMF Complex III Q_i_ site was generated using LigPlot+.^88^

#### Quantification and Statistical Analysis

Particle numbers and reconstruction statistics for cryo-EM analyses are provided in Table S5. Details of the complexome profiling and mass spectrometry analyses, including sample numbers and associated quantitative analyses, are provided in the corresponding Method Details and figure legends.

## Supplementary Material

1 **Document S1.** Figures S1–S4 and Tables S5–S8

2 **Table S1.** Complexome protein group data for LtPMF (Tab A), TbPCF (Tab B), and TbBSF (Tab C), related to Figures 1–3

3 **Table S2.** Co-migration profiles of MitoCarta proteins (Tab A) and annotated complexes (Tabs B-K) across LtPMF, TbPCF, and TbBSF, related to Figures 2–3

4 **Table S3.** Manual annotation of mitochondrial complexes, related to Figures 2 and 3

5 **Table S4.** Apparent molecular mass assignments for BN-PAGE slices, related to Figures 1–3

## Figures and Tables

**Figure 1.**
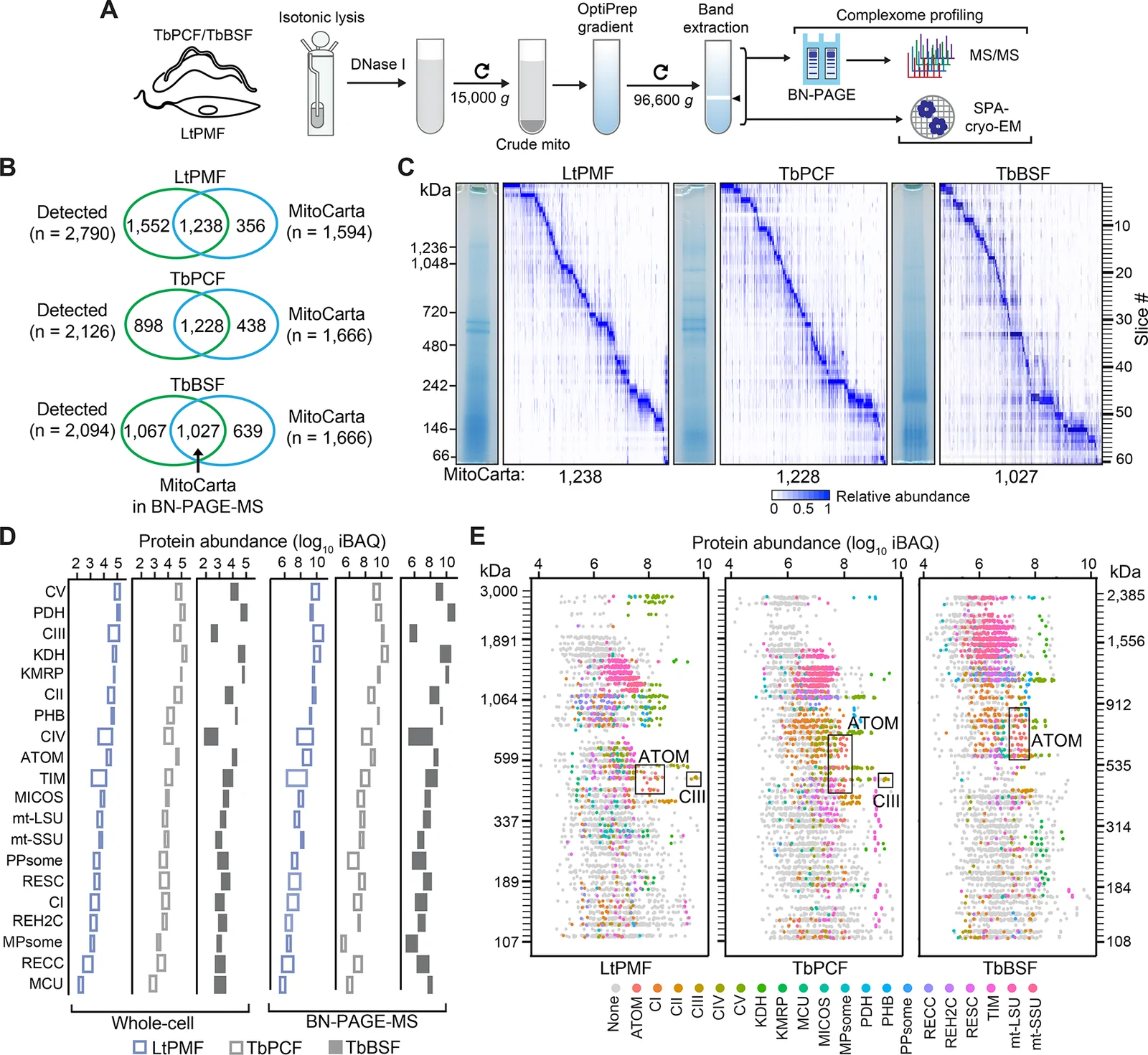
Quantitative complexomes of model kinetoplastids (A) Schematic of the complexome characterization workflow. SPA-cryo-EM, single-particle analysis cryo-electron microscopy. (B) The total number of proteins detected in BN-PAGE-MS and MitoCarta proteins among them. (C) Coomassie-stained BN-PAGE gels aligned with heatmaps showing relative abundances of identified mitochondrial proteins (columns). For each protein, iBAQ values were normalized to the maximum signal across 59 gel slices from the corresponding lane (slice 1 excluded). The number of MitoCarta proteins detected per dataset is indicated at the bottom. (D) Boxplots showing the abundance distributions (25th–75th percentile) of proteins assigned to 20 annotated complexes (Table S3). Complexes are ordered by their abundance in the LtPMF whole-cell lysates. (E) Abundance of MitoCarta proteins (X-axis) is shown for each BN-PAGE slice (Y-axis; slice 1 excluded). Proteins belonging to the 20 major annotated complexes are color-coded; proteins not assigned to any complex are shown in gray. For visualization, only slices with relative abundance ≥0.5 are displayed. Apparent molecular mass markers are shown for LtPMF and TbPCF (left axis) and TbBSF (right axis) (Table S4).

**Figure 2.**
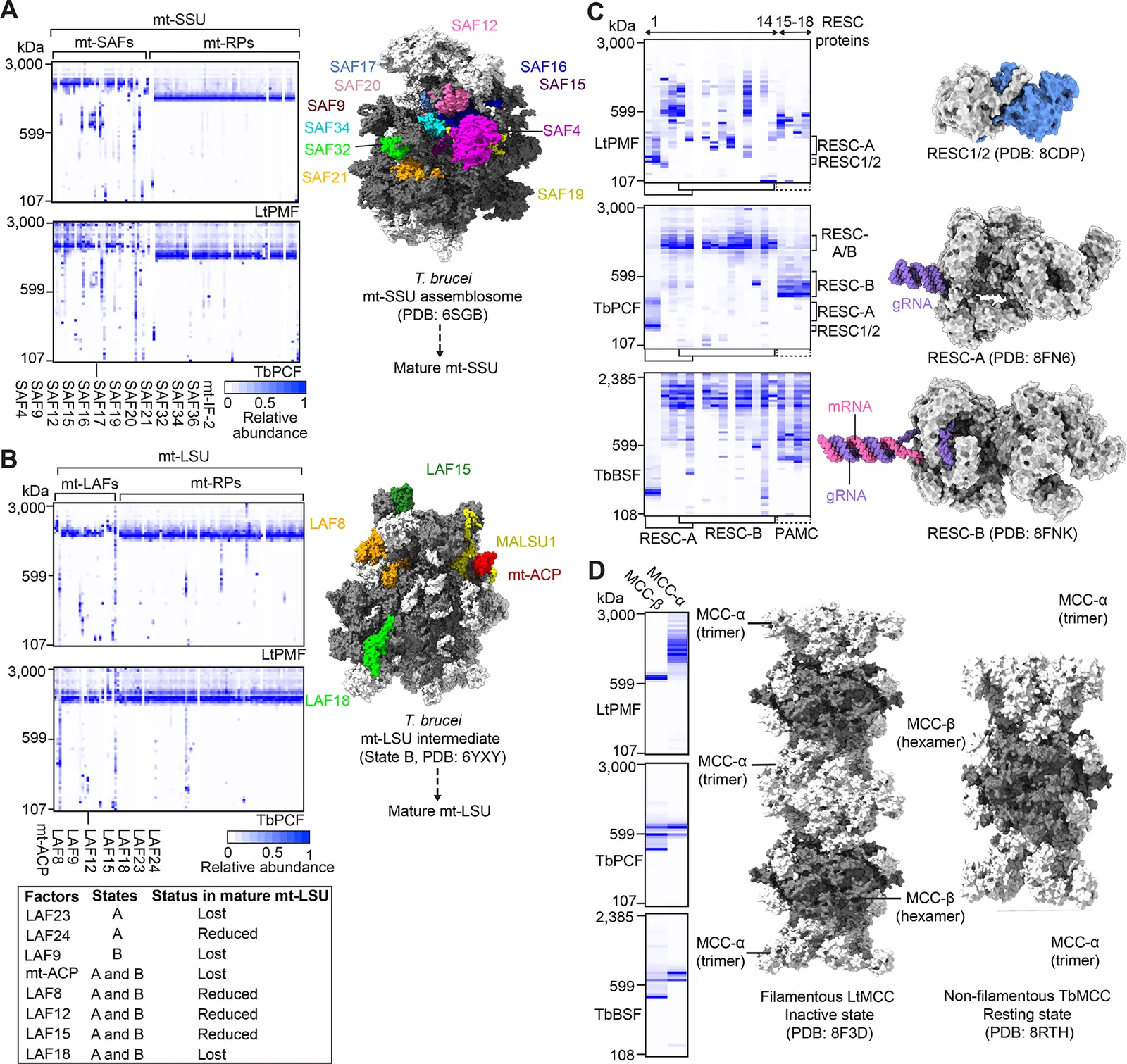
Integrative complexome and structural analyses reveal dynamics and species-specific features of mitochondrial complexes (A) BN-PAGE profiles of the small ribosomal subunits (mt-SSUs) from LtPMF and TbPCF. Cryo-EM structure of the TbPCF mt-SSU “assemblosome.” The indicated small-subunit assembly factors (mt-SAFs) dissociate during mt-SSU maturation. Ribosomal proteins are shown in white, retained mt-SAFs in gray, and dissociating factors in color. (B) BN-PAGE profiles of the large ribosomal subunit (mt-LSU) from LtPMF and TbPCF. The box panel shows the assembly factors at precursor states and loss or retention during maturation. Cryo-EM structure of a TbPCF mt-LSU assembly intermediate (State B). The large-subunit assembly factors (mt-LAFs) that dissociate during biogenesis are listed at the bottom. mt-RPs are shown in white, retained mt-LAFs in gray, and dissociating factors in color. (C) BN-PAGE profiles of the RESC1/2 (first two lanes), RESC-A, RESC-B, and polyadenylation mediator (PAMC) complexes. The potential higher-order RESC-A/B assembly is indicated. Cryo-EM structures of the reconstituted RESC1/2 heterodimer^27^ and endogenous TbPCF RESC-A and RESC-B complexes^8^ are shown. (D) BN-PAGE profiles of 3-methylcrotonyl-CoA carboxylase (MCC) from LtPMF, TbPCF, and TbBSF. Cryo-EM structures of filamentous MCC from LtPMF^10^ and non-filamentous MCC from TbPCF^9^ are shown. MCC-α subunits are shown in white and MCC-β subunits in gray.

**Figure 3.**
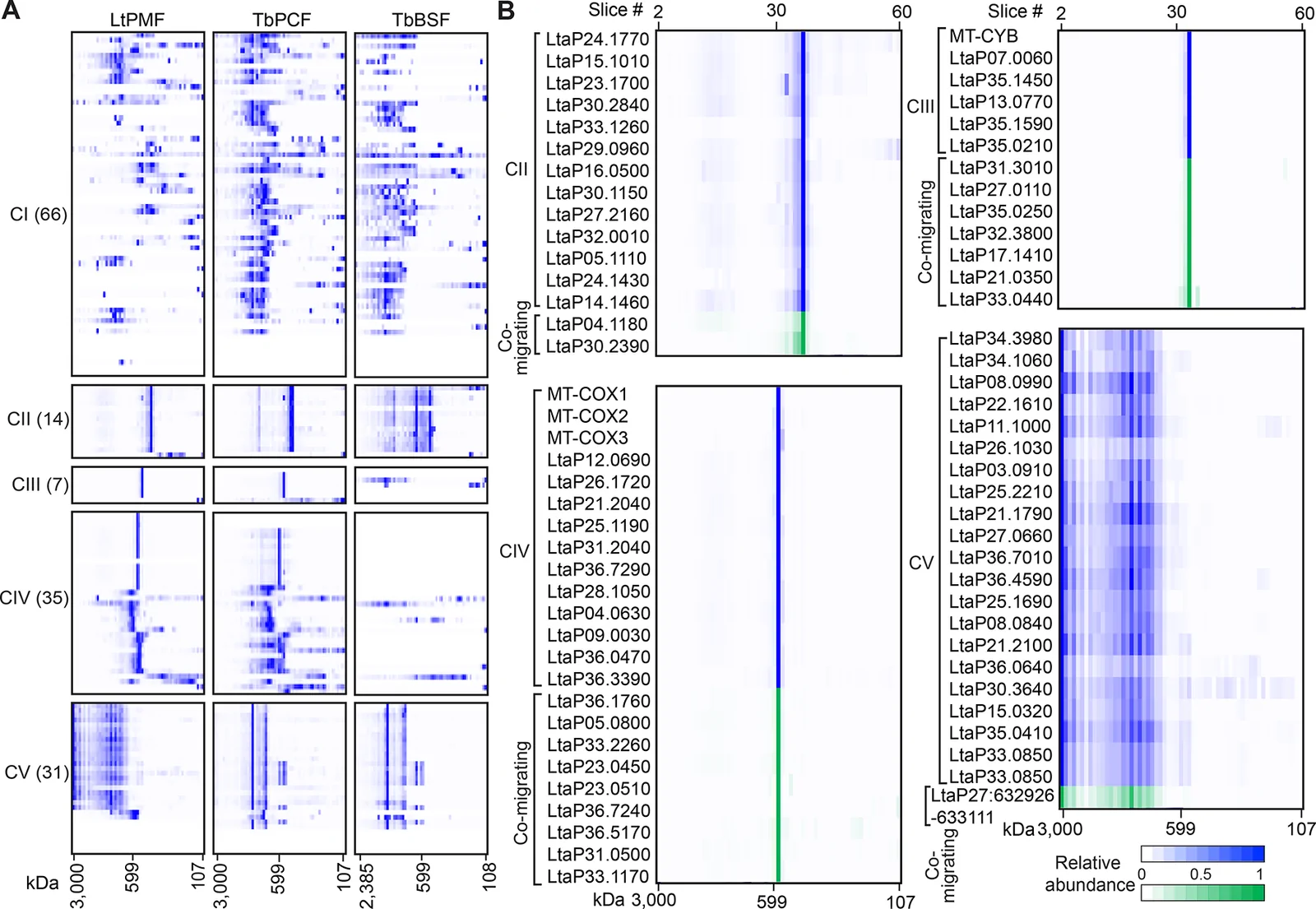
Complexome profiling defines the composition and heterogeneity of respiratory complexes (A) BN-PAGE profiles of respiratory complexes I–V (CI–CV) across kinetoplastid models. The total number of subunits in each complex is indicated. (B) BN-PAGE profiles of conserved and lineage-specific components of LtPMF respiratory complexes II–V. Relative abundances of proteins shown in blue were used as “baits,” and proteins shown in green were predicted as co-migrating interactors.

**Figure 4.**
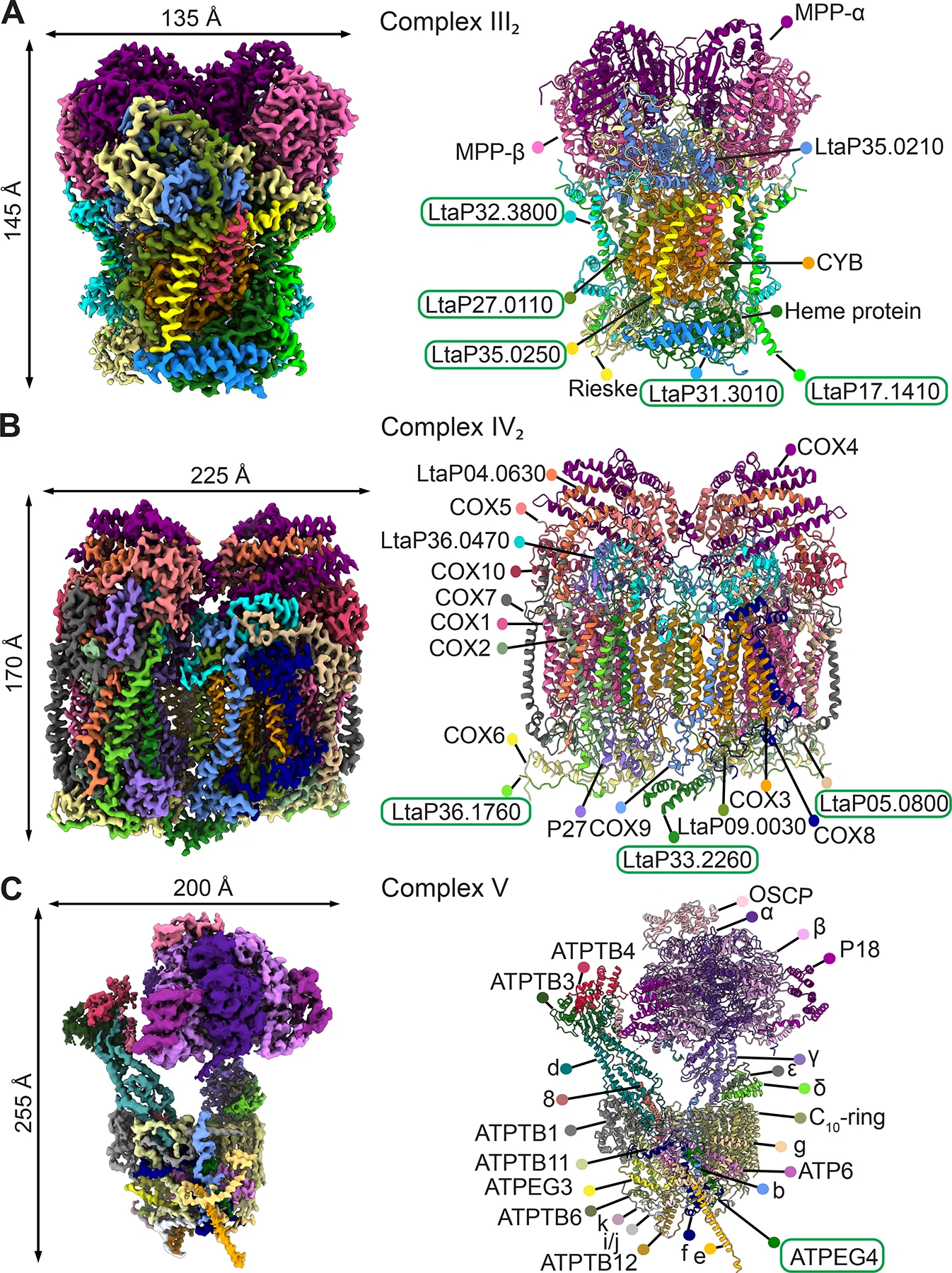
Cryo-EM structures of LtPMF respiratory complexes III2, CIV2, and CV (A–C) Cryo-EM density maps (left) and corresponding structural models (right) of *L. tarentolae* complex III_2_, complex IV_2_, and ATP synthase (CV). Density maps are colored by subunit according to the corresponding models. Subunits identified directly in the cryo-EM maps and validated by complexome profiling are indicated by green boxes. For CV, the F_0_-region subunits were built and refined against the corresponding cryo-EM density, and the peripheral-stalk subunits were predicted using AlphaFold 3, fitted into the corresponding density, and subjected to restrained real-space refinement. The F_1_/central-stalk region is represented by a model fitted into the rotation 1 (Rot1) density but not subjected to map-guided rebuilding or refinement.

**Figure 5.**
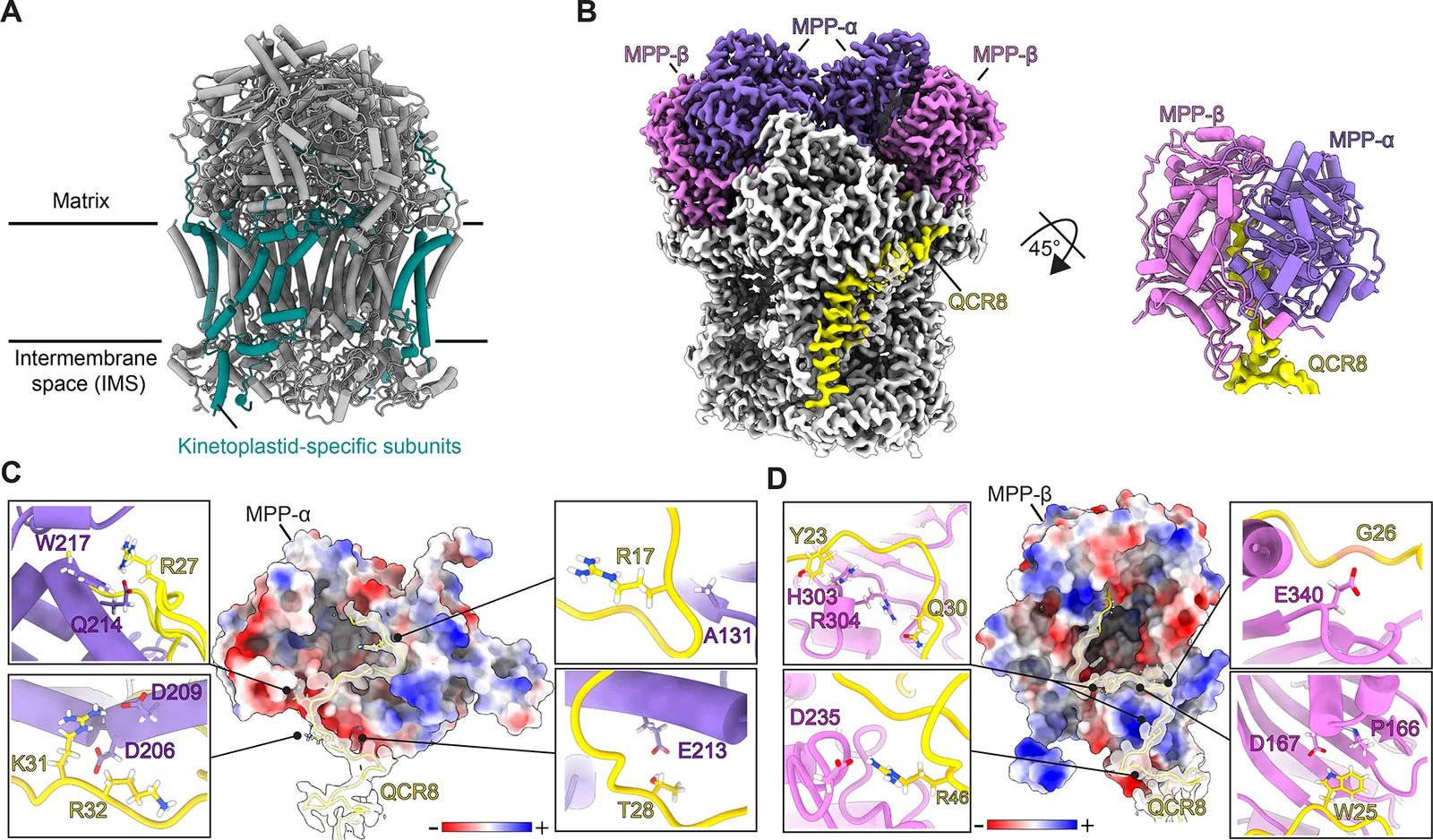
Cryo-EM structure of CIII2 reveals clade-specific MPP–QCR8 interactions and the Qi-site inhibitor–binding pocket (A) Subunit organization of LtPMF CIII_2_ highlighting kinetoplastid-specific components (teal). (B) Cryo-EM density of the MPP heterodimer showing the N-terminal extension of the QCR8 subunit occupying the substrate-binding cavity. MPP-*α*, violet; MPP-*β*, pink; QCR8, gold. (C) Surface charge representation of MPP-*α* interacting with the QCR8 N-terminal extension. Close-up views show complementary electrostatic contacts. (D) Surface charge representation of MPP-*β* interacting with the QCR8 N-terminal extension, with close-up views of local interfaces.

**Figure 6.**
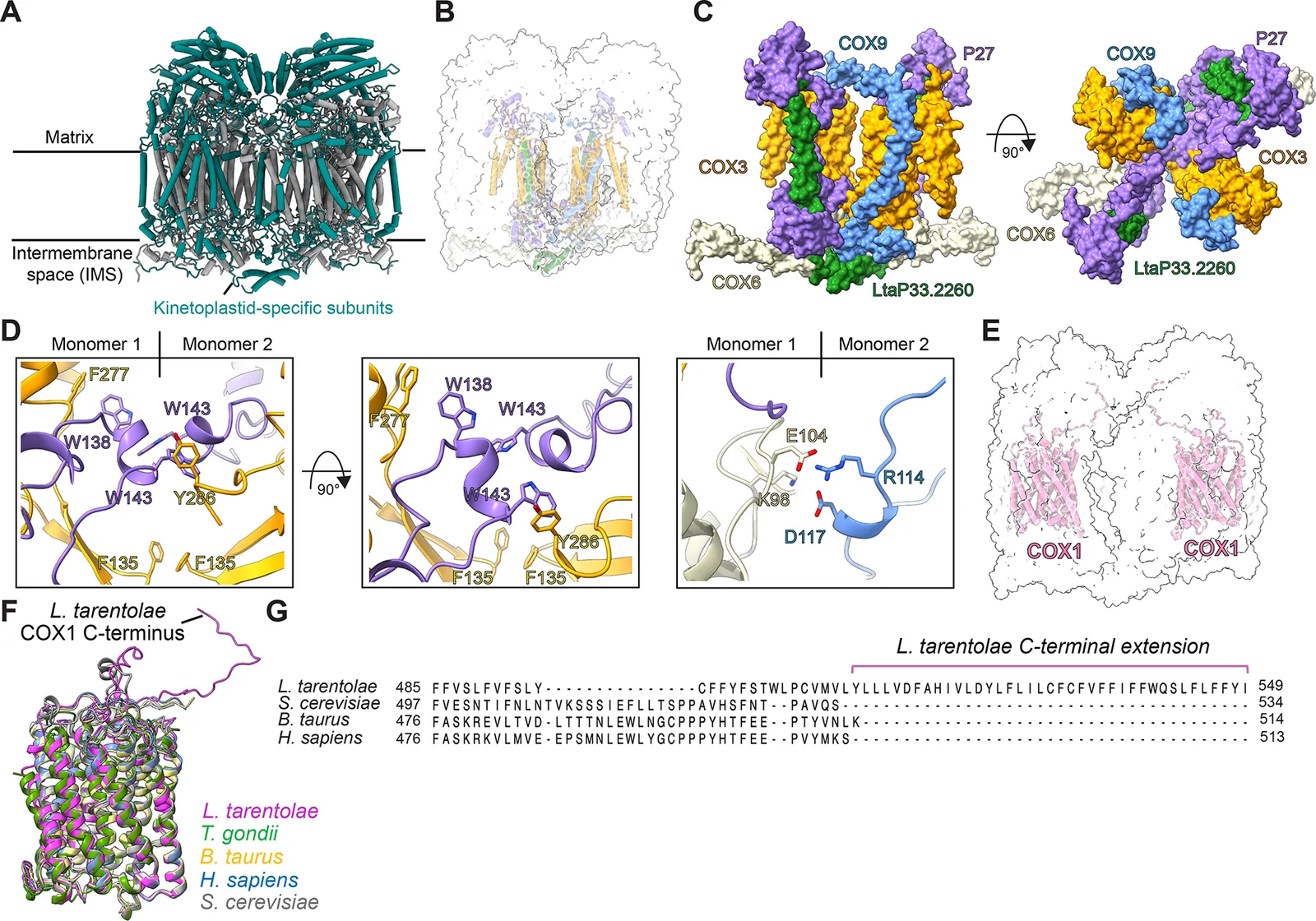
Cryo-EM structure of Complex IV2 from L. tarentolae (A) Cryo-EM structure of LtPMF CIV_2_ showing clade-specific architectural elaborations. Subunits unique to kinetoplastids are highlighted in teal. (B-C) Surface representation of the CIV_2_ dimer highlighting major interacting subunits at the dimer interface. COX3, orange; COX9, medium blue; LtaP33.2260, green; P27, purple; COX6, khaki. (D) Close-up views of CIV_2_ dimer contacts. Dimer stabilization is mediated primarily by π-stacking and complementary electrostatic interactions between interface residues. (E) CIV_2_ rendered as a transparent surface with COX1 shown in cylinder representation to illustrate its extended C-terminal region. (F) Structural superposition of COX1 from L. tarentolae (LtPMF), S. cerevisiae (PDB: 8DH6), B. taurus (PDB: 7W3E), T. gondii (PDB: 9I4Y), and H. sapiens (PDB: 5Z62). The extended C-terminus of L. tarentolae COX1 is indicated. (G) Multiple sequence alignment of the COX1 C-terminal region from L. tarentolae, S. cerevisiae, B. taurus, and H. sapiens, highlighting the C-terminal extension unique to L. tarentolae. This extension may contribute to stabilization of the enlarged CIV monomer.

**Figure 7.**
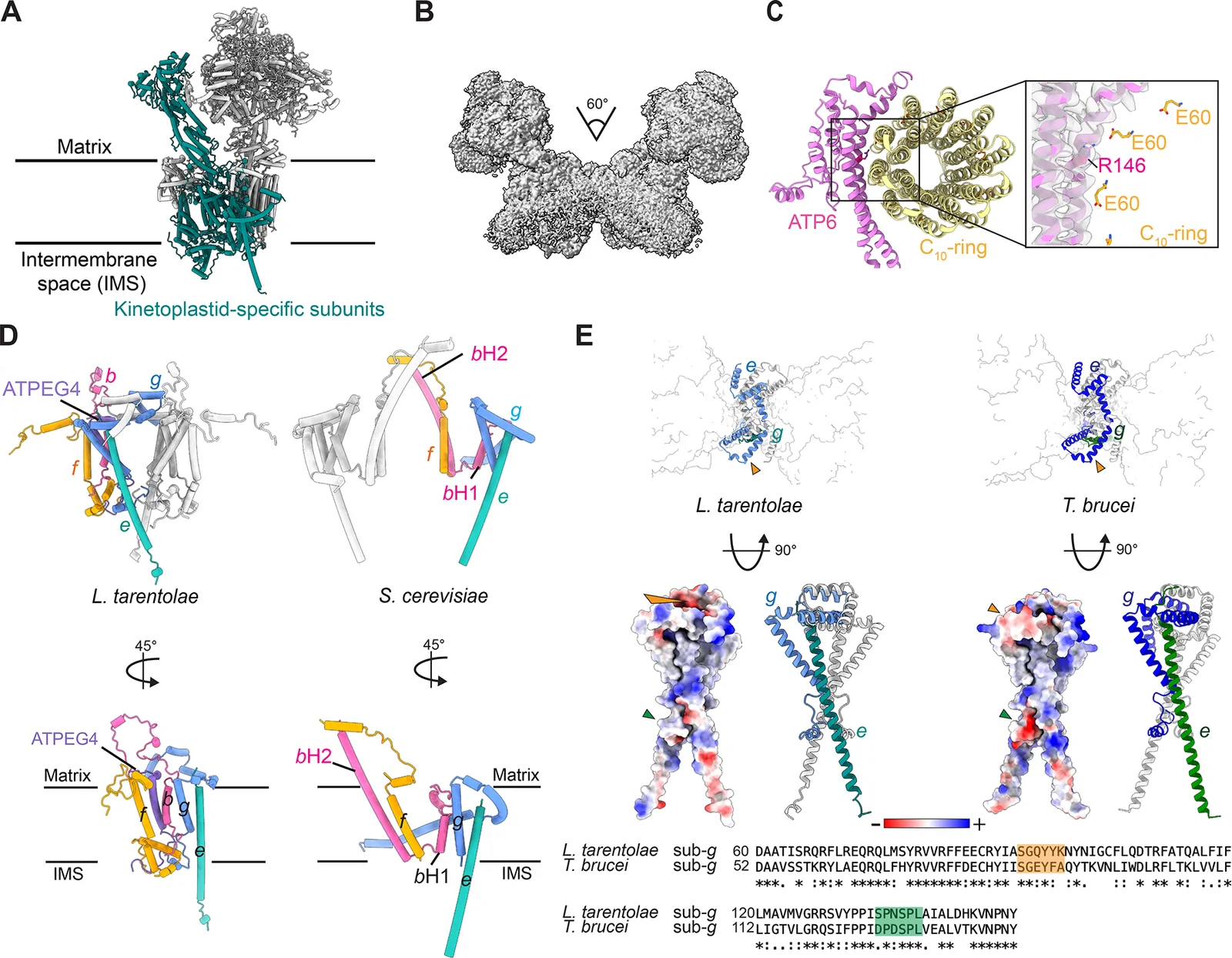
Cryo-EM structure of CV (ATP synthase) reveals a compact dimer architecture and lineage-specific peripheral stalk organization (A) Cryo-EM structure of LtPMF CV highlighting clade-specific structural elements. All kinetoplastid-specific subunits are located within the peripheral-stalk and F_*0*_ membrane regions and are shown in teal. The F_1_/central-stalk region is represented by a model fitted into the Rot1 cryo-EM density but not subjected to map-guided rebuilding or refinement. (B) Cryo-EM density map of the LtPMF CV dimer, showing a compact architecture with an intermonomer angle of approximately 60° between the two F_1_–C_10_-ring subcomplexes. This configuration contrasts with the wider-angle dimers observed in animals and fungi and resembles that reported for *T. brucei*. (C) The F_o_ domain contains a C_10_-ring coupled to the kDNA-encoded ATP6 subunit (pink). A close-up view shows the conserved electrostatic pair between Arg146 (ATP6) and Glu60 (C_10_-ring), mediating proton transfer between the half-channels. This interaction is functionally analogous to the aArg176–cGlu59 pair in *S. cerevisiae*. In *T. brucei*, the corresponding Arg on ATP6 contacts cGlu102, underscoring conservation of the proton-handoff mechanism. (D) The peripheral stalk and transmembrane dimer interface differ markedly from those of opisthokont ATP synthases. In LtPMF, subunit b contains a single transmembrane helix (bH1), which anchors subunits e and g. The long bH2 helix characteristic of yeast and mammalian ATP synthases is absent; instead, ATPEG4 occupies this position, providing stalk stabilization unique to kinetoplastids. (E) Electrostatic surface representations of subunits e and g from LtPMF and *T. brucei*. Both contribute to higher-order ATP synthase oligomerization, but LtPMF subunit g exhibits a more positively charged surface (arrowheads; see sequence alignment), suggesting species-specific tuning of dimer and oligomer stability. See also Table S8.

**Table:** KEY RESOURCES TABLE

REAGENT OR RESOURCE	SOURCE	IDENTIFIER
**Chemicals, peptides, and recombinant proteins**		
Brain Heart Infusion (BHI) broth	Sigma	Cat# 53286
Hemin	Sigma	Cat#16009-13-5
OptiPrep media	Cosmo Bio USA Inc.	Cat#AXS1114542
Phenylmethanesulfonyl fluoride (PMSF)	Millipore Sigma	Cat#P7626–250MG
cOmplete, Mini, EDTA-free Protease Inhibitor Cocktail	Roche	Cat#4693159001
Fetal calf serum	Biotechne	Cat#S10350H
Sorbitol	Thermo Fisher Scientific	Cat#03640436
Turbo DNase	Invitrogen	Cat#AM2239
Benzonase nuclease	Sigma	Cat#70-664-3
Acetonitrile	Sigma	Cat#75-05-8
Ammonium bicarbonate	Sigma	Cat#1066-33-7
**Critical commercial assays**		
Pierce^™^ BCA Protein Assay Kit	Thermo Fisher Scientific	Cat#PI23225
**Deposited data**		
Cryo-EM structure of *Leishmania tarentolae* respiratory complex III (cytochrome bc1 complex)	This paper	PDB: 9Z9K / EMD-73948
Cryo-EM structure of *Leishmania tarentolae* respiratory complex IV (cytochrome c oxidase dimer)	This paper	PDB: 9ZAK / EMD-73966
Cryo-EM structure of *Leishmania tarentolae* respiratory complex V (ATP synthase) F_o_ dimer membrane region	This paper	PDB: 9ZB4 / EMD-73974
Cryo-EM structure of *Leishmania tarentolae* respiratory complex V (ATP synthase) peripheral stalk	This paper	PDB: 9ZC4 / EMD-74030
Cryo-EM structure of *Leishmania tarentolae* respiratory complex V (ATP synthase) F_o_ monomer membrane region	This paper	PDB: 9ZC5 / EMD-74031
Cryo-EM density of *Leishmania tarentolae* respiratory complex V (ATP synthase) F_1_/central-stalk	This paper	EMD-78449
Cryo-EM density of *Leishmania tarentolae* respiratory complex V (ATP synthase) dimer	This paper	EMD-78468
Crystal structures of yeast mitochondrial processing peptidase	Taylor et al.^36^	PDB: 1HR6
Cryo-EM structure of the *Arabidopsis thaliana* I+III_2_ supercomplex	Klusch et al.^40^	PDB: 8BQ6
Cryo-EM structure of *Toxoplasma gondii* respiratory chain supercomplex	MacLean et al.^38^	PDB: 9G9T, 9I4Y
Cryo-EM structure of human mitochondrial respiratory megacomplex I(2)III(2)IV(2).	Guo et al.^39^	PDB: 5XTE
Cryo-EM structure of vascular plants respiratory complex III(2), complex IV	Maldonado et al.^43^	PDB: 7JRP, 7JRO
Cryo-EM structure of the yeast respiratory supercomplex	Hartley et al.^66^	PDB: 6HU9
Cryo-EM structure of yeast respiratory supercomplex III2IV	Rathore et al.^37^	PDB: 6GIQ
Cryo-EM structure of yeast CIV	Godoy et al.	PDB: 8DH6
Cryo-EM structure of human CIV	Zong et al.^44^	PDB: 5Z62
Crystal structure of bovine CIV	Shimada et al.^45^	PDB: 7W3E
Cryo-EM structure of *Trypanosoma brucei* ATP synthase F_1_F_*o*_ dimer	Gahura et al.^12^	PDB: 8AP6
Cryo-EM structure of the dimeric Fo region of yeast ATP synthase	Guo et al.^54^	PDB: 6B2Z
Cryo-EM structure of *Trypanosoma brucei* mt-SSU assemblosome	Saurer et al.^22^	PDB: 6SGB
Cryo-EM structure of *Trypanosoma brucei* mt-LSU intermediate (State B)	Jaskolowski et al.^25^	PDB: 6YXY
Cryo-EM structure of *Trypanosoma brucei* RESC1/2	Dolce et al.^27^	PDB: 8CDP
Cryo-EM structure of *Trypanosoma brucei* RESC-A	Liu et al.^7^	PDB: 8FN6
Cryo-EM structure of *Trypanosoma brucei* RESC-B	Liu et al.^7^	PDB: 8FNK
Cryo-EM structure of *Trypanosoma brucei* MCC	Plaza-Pegueroles et al.^9^	PDB: 8RTH
Cryo-EM structure of *Leishmania tarentolae* MCC	Hu et al.^10^	PDB: 8F3D
*Leishmania tarentolae* Parrot Tar II proteome	Goto et al.^78^	TriTrypDB: https://tritrypdb.org/tritrypdb/app
Mass spectra and the protein sequence databases used for searches	This paper	MassIVE: MSV000102789
Blue native PAGE images	This paper	Mendeley Data: https://doi.org/10.17632/d5ywdrwzkn.2
**Experimental models: Organisms/strains**		
*Leishmania tarentolae* (UC) strain	Larry Simpson, UCLA	N/A
*Trypanosoma brucei* Lister 427 procyclic form	Larry Simpson, UCLA	N/A
*Trypanosoma brucei* Lister 427 bloodstream form	Larry Simpson, UCLA	N/A
**Software and algorithms**		
EPU	Thermo Fisher Scientific	https://www.thermofisher.com/
cryoSPARC (v.4.7.0)	Punjani et al.^76^	https://cryosparc.com
UCSF ChimeraX (v.1.6)	Goddard al.^77^	https://www.cgl.ucsf.edu/chimerax
Coot (v.0.9.6)	Emsley et al.^81^	https://www2.mrc-lmb.cam.ac.uk/personal/pemsley/coot
Phenix (v.1.21.2–5419)	Adams et al.^79^	https://www.phenix-online.org
ModelAngelo (version 1.014)	Jamali et al.^35^	https://github.com/3dem/model-angelo
AlphaFold 3	Abramson et al.^73^	https://alphafoldserver.com
RosettaLigand	Meiler et al.^84^, Davis et al.^85^	https://github.com/RosettaCommons/rosetta
Corina	Corina online	https://demos.mn-am.com/corina.html
AmberTools	Case et al.^86^	https://ambermd.org/AmberTools.php
PatchDock	Schneidman-Duhovny et al.^87^	https://bioinfo3d.cs.tau.ac.il/PatchDock/
Ligplot^+^	Wallace et al.^88^	https://www.ebi.ac.uk/thornton-srv/software/LigPlus
RING 4.0	Del Conte et al.^83^	https://ring.biocomputingup.it
ISOLDE (v1.12)	Croll et al.^80^	https://tristanic.github.io/isolde/
**Other**		
Quantifoil R1.2/1.3 300 mesh, Cu grids	Quantifoil	Cat#Q3100CR1.3

## Highlights

- System-wide atlases of mitochondrial complexes in two kinetoplastid model organisms
- Cryo-EM structures of *L. tarentolae* respiratory complexes CIII_2_, CIV_2_, and CV
- Nine previously unrecognized subunits assigned by cryo-EM
- Five mitochondrially encoded proteins visualized within CIII_2_, CIV_2_, and CV structures
