# Supplementary material for: Conserved and divergent mitochondrial assemblies in kinetoplastid parasites
Source: Mol Cell. Author manuscript; Available in PMC 2026 Oct 2. (PMC13631411; doi:10.1016/j.molcel.2026.08.031)
Supplement: Supplementary file 1 [file NIHMS2209500-supplement-1.pdf]

Supplemental information

Conserved and divergent mitochondrial assemblies in kinetoplastid parasites

Md Solayman, Yi-Ting Liao, Sarah E. Calvo, Andres F. Vacas, Malvina Papanastasiou, Keith D. Rivera, DR Mani, Brian W. Boyle, Felicia G. Deng, MitoCarta Tree of Life Consortium, Namrata D. Udeshi, Vamsi K. Mootha, Inna Aphasizheva, Luke H. Chao, and Ruslan Aphasizhev

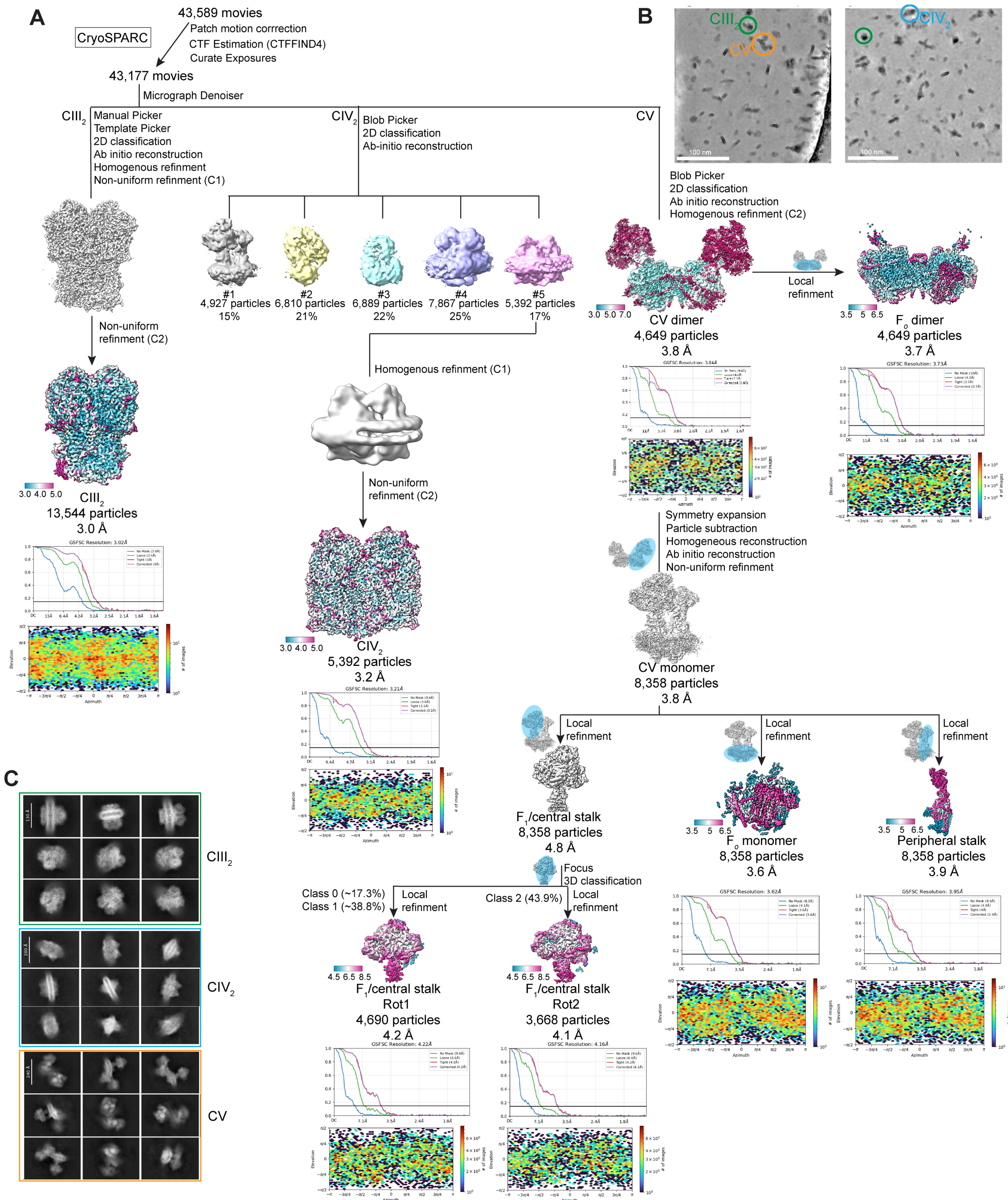

Figure S1. **Cryo-EM data processing workflow, related to Figures 4–7.**
(A) Overview of the cryo-EM data processing pipeline for $CIII_2$, CIV_2, and CV (see Table S5). The flowchart summarizes particle selection, classification, and refinement steps performed in cryoSPARC. Local resolution maps are shown for $CIII_2$, CIV_2, CV dimer and the CV subcomplexes (F_o dimer membrane region, F_1/central stalk Rot1, F_1/central stalk Rot2, F_o monomer membrane region, and peripheral stalk), together with their corresponding gold-standard Fourier shell correlation (GSFSC) curves and particle Euler angle distributions. The composite CV monomer

map presented in Figure 4C was assembled from the locally refined maps of the F_1/central stalk in Rot1, F_o monomer membrane region, and peripheral stalk. The corresponding composite structural model is shown together with the map in Figure 4C and independently in Figure 7A. The F_1/central-stalk component of this model was fitted into the Rot1 density but was not subjected to map-guided rebuilding or refinement. The CV dimer map is shown in Figure 7B.
(B) Representative micrographs with highlighted particles corresponding to $CIII_2$ (green), CIV_2 (orange), and CV (blue).
(C) Representative 2D class averages for $CIII_2$ (top), CIV_2 (middle), and CV (bottom).

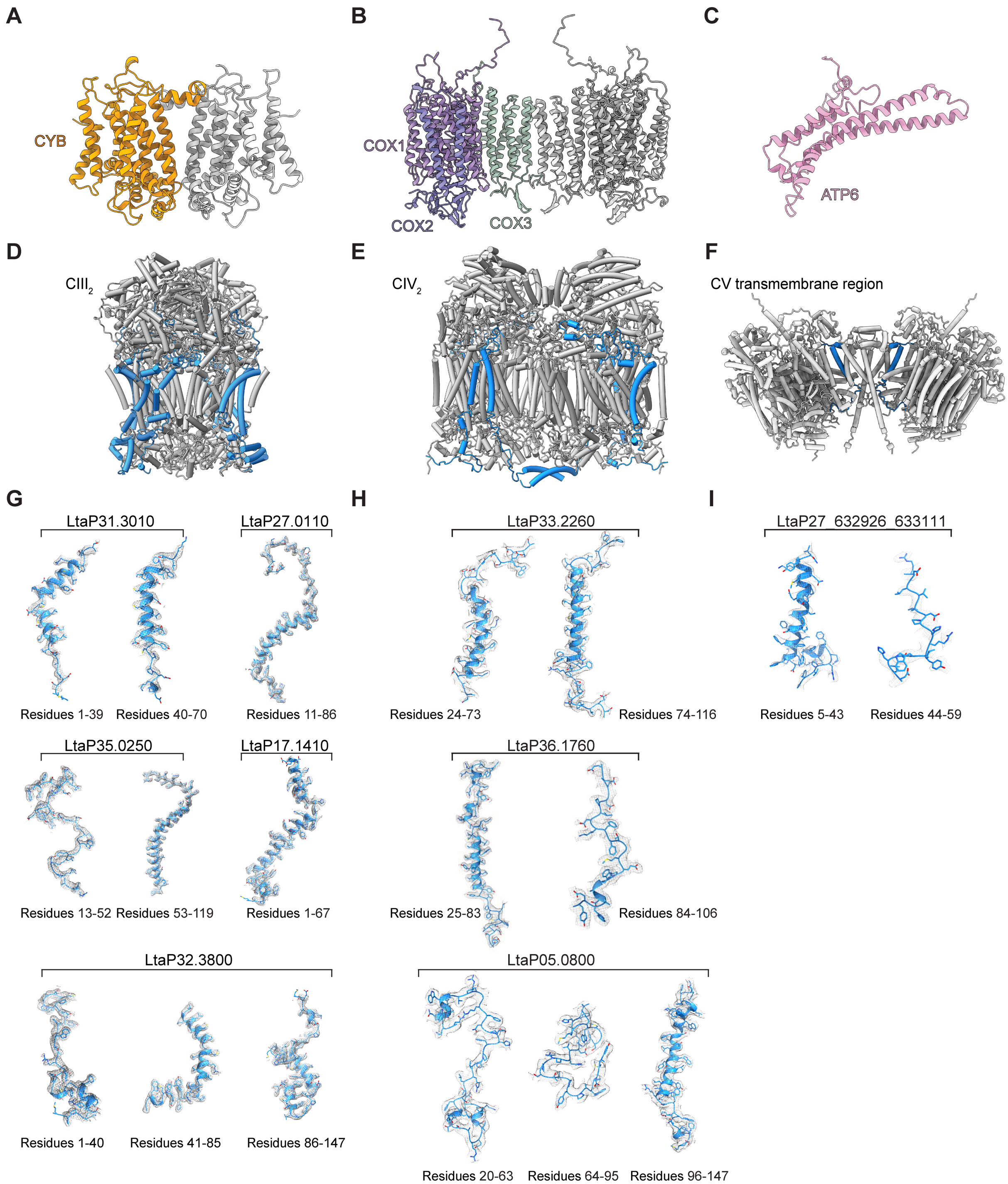

Figure S2. **Mitochondrially encoded subunits and validation of newly identified proteins in the cryo-EM models, related to Figures 4–7.**

(A-C) Atomic models of mitochondrially (kDNA) encoded subunits visualized in the cryo-EM reconstructions: (A) cytochrome b (CYB) in $CIII_2$, (B) COX1–3 in CIV_2, and (C) ATP6 in CV.

(D-F) Spatial positions of newly identified subunits (blue) within the LtPMF respiratory complexes: (D) $CIII_2$, (E) CIV_2, and (F) CV.

(G-I) Representative cryo-EM densities (gray mesh) corresponding to newly identified subunits, shown with their fitted atomic models (blue ribbon): (G) $CIII_2$, (H) CIV_2, and (I) CV.

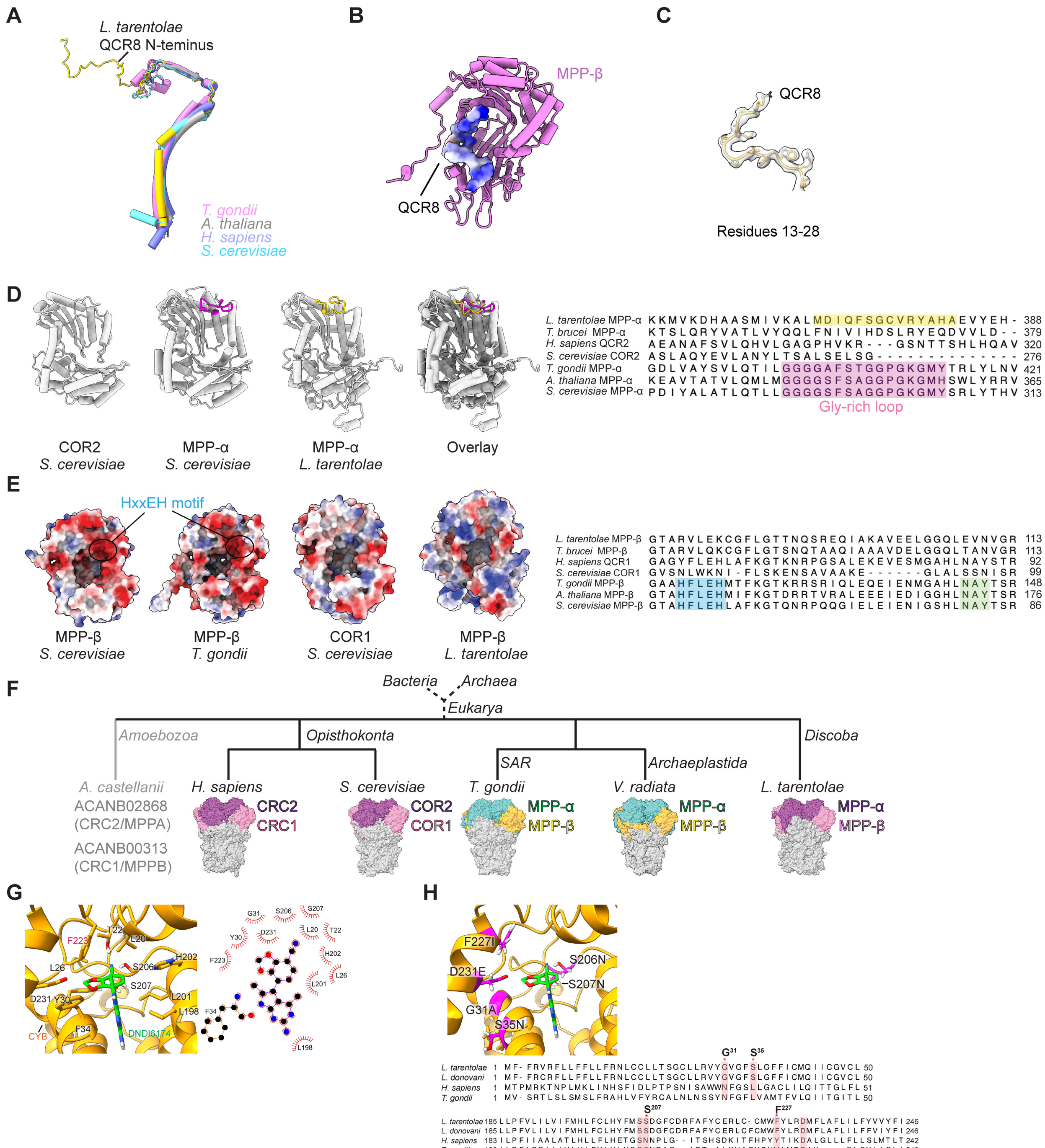

Figure S3. Structural features and functional divergence of the MPP heterodimer, related to Figure 5.

(A) Structural overlay of QCR8 from *A. thaliana* (PDB: 8BQ6),[40] *H. sapiens* (PDB: 5XTE),[39] *S. cerevisiae* (PDB: 6GIQ),[37] *T. gondii* (PDB: 9G9T),[38] and *L. tarentolae* (LtPMF), showing an N-terminal extension unique to kinetoplastids.

(B) Surface electrostatic representation of QCR8 (residues 13–28) and MPP-*β*. The positively charged N-terminal extension of QCR8 occupies the MPP*α*/*β* cavity.

(C) Representative cryo-EM density for the N-terminal extension of QCR8 (residues 13–28).
(D) Comparison of MPP-*α* structures from *S. cerevisiae* and LtPMF, along with COR2 from *S. cerevisiae*.[36,37] Bottom, multiple sequence alignment of MPP-*α* orthologs from *T. brucei*, *S. cerevisiae*, *A. thaliana*, *T. gondii*, and LtPMF, together with COR2 (*S. cerevisiae*) and QCR2 (*H. sapiens*). As in opisthokont COR2/QCR2, LtPMF MPP-*α* lacks the canonical substrate-binding Gly-rich loop (magenta).
(E) Surface electrostatic representations of MPP-*β* homologs from *S. cerevisiae*, *T. gondii*, and LtPMF, along with COR1 (*S. cerevisiae*).[36-38] Bottom, multiple sequence alignment of MPP-*β* orthologs from *T. brucei*, *S. cerevisiae*, *A. thaliana*, *T. gondii*, and LtPMF, together with COR1 (*S. cerevisiae*) and QCR1 (*H. sapiens*). LtPMF MPP-*β* lacks the catalytic HxxEH motif (cyan) and the NAY motif (green), consistent with loss of proteolytic activity.
(F) Phylogenetic distribution of MPP/COR/CRC homologs across eukaryotes.[38,43,66] LtPMF MPP*α*/*β* (purple/pink) retain the MPP structural scaffold but lack substrate-binding and catalytic motifs, analogous to opisthokont COR/CRC. In contrast, active MPPs from *T. gondii* and *V. radiata* (green/yellow) retain these features.
(G) Modeled binding mode of the antileishmanial compound DNDI-6174 within the Q_i site of cytochrome b, obtained by molecular docking. Right: schematic depiction of ligand–protein contacts. Phe223 forms a π–π stacking interaction with the aromatic ring of DNDI-6174, while Phe34, Tyr30, Leu201, and Leu198 contribute to a predominantly nonpolar pocket that stabilizes the ligand. Hydrophobic contacts are shown as red spokes.
(H) Resistance-associated cytochrome *b* substitutions identified in DNDI-6174-resistant *L. donovani* lines mapped onto the molecular-docking model and shown in magenta. Right, multiple sequence alignment of the Q_i site in cytochrome b from LtPMF, *L. donovani*, *H. sapiens*, and *T. gondii*, with resistance-associated positions marked by stars. The pattern indicates that DNDI-6174 is unlikely to inhibit cytochrome b in mammals or apicomplexans.

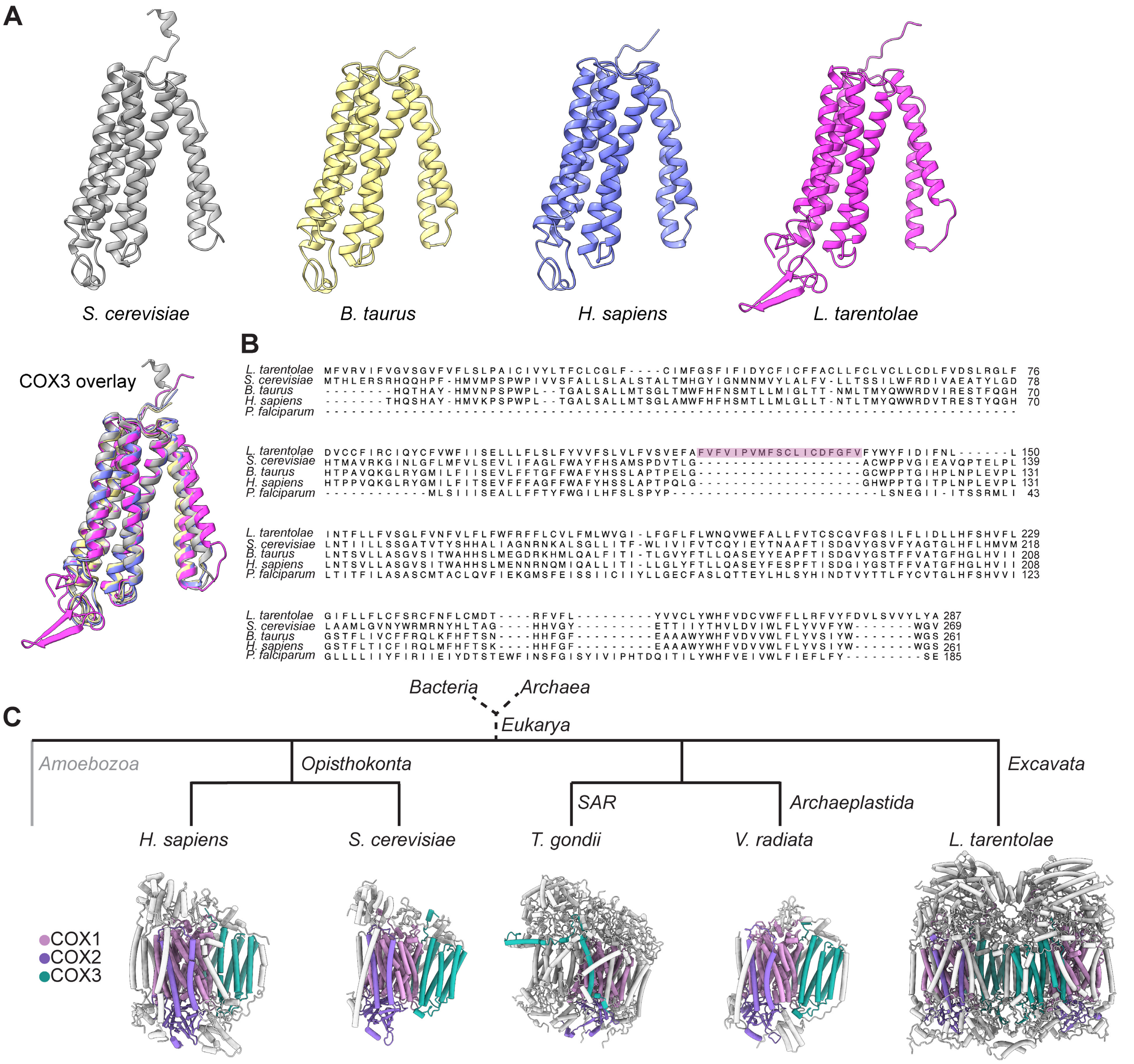

Figure S4. Structural comparison of COX3 and evolutionary diversification of Complex IV, related to Figure 6.

(A) Structural overlay of COX3 from *S. cerevisiae* (PDB: 8DH6), *B. taurus* (PDB: 7W3E), *H. sapiens* (PDB: 5Z62), and *L. tarentolae* (LtPMF).[44,45]

(B) Multiple sequence alignment of COX3 from *S. cerevisiae*, *B. taurus*, *H. sapiens*, *P. falciparum*, and *L. tarentolae* (LtPMF). In LtPMF, COX3 contains additional β-sheet extensions (highlighted in magenta) that contribute to the CIV_2 dimer interface.

(C) Phylogenetic comparison of Complex IV architecture across eukaryotes: *H. sapiens* (PDB: 5Z62), *S. cerevisiae* (PDB: 8DH6), *V. radiata* (PDB: 7JRO), *T. gondii* (PDB: 9I4Y), and *L. tarentolae* (LtPMF).[38,43,44] COX1, COX2, and COX3 are shown in pink, purple, and sea green, respectively. Although overall CIV assembly varies widely among eukaryotic lineages, Discoba representatives such as *L. tarentolae* exhibit a reinforced CIV_2 dimer interface formed by kinetoplastid-specific subunits, reflecting a lineage-specific mechanism of Complex IV stabilization.

Table S5. Data collection, processing, model refinement, and validation statistics for Complex III_2, Complex IV_2, and Complex V (ATP synthase), related to Figure S1.

	Complex III	Complex IV	ATP synthase F_o dimer membrane region	ATP synthase peripheral stalk	ATP synthase F_o monomer membrane region
PDB	9Z9K	9ZAK	9ZB4	9ZC4	9ZC5
EMDB	73948	73966	73974	74030	74031
**Data collection**					
Cryo-EM equipment			Titan Krios		
Voltage (kV)			300		
Magnification (nominal)			165000		
Detector			K3		
Dose rate (e–/Å^2 per second)			~22.6		
Pixel size (Å/pixel)			0.736		
Electron exposure (e–/Å^2)			~52		
Exposure time (s)			2.3		
Defocus range (μm)			-0.6 to -1.8		
Number of micrographs			43,589		
**Data processing**					
Software			cryoSPARC		
Symmetry imposed	C2	C2	C2	C1	C1
Final particle images (no.)	13,544	5392	4649	8358	8358
Map resolution (Å, FSC=0.143)	3.02	3.21	3.73	3.95	3.62
**Atomic modeling** software	ModelAngelo, AlphaFold 3 and ChimeraX	ModelAngelo and ChimeraX	ModelAngelo and ChimeraX	AlphaFold 3 and ChimeraX	ModelAngelo and ChimeraX
**Refinement** software			Coot, ISOLDE and Phenix		
Resolution of unmasked reconstruction (Å, FSC=0.5)	3.4	3.7	4.4	12,6	4.3
Resolution of masked reconstruction (Å, FSC=0.5)	3.3	3.4	4.0	7.3	3.9
Correlation coefficient (CC_{mask})	0.89	0.89	0.84	0.74	0.84
Map sharpening B-factor (Å^2)	-43.72	-17.52			
Model composition					
Non-hydrogen atoms	41,040	53,314	41,236	5,949	20,618
Protein residues	5,116	6,448	5,124	756	2,562
Ligands	4	6			
B factors (Å^2)	min/max/mean	min/max/mean	min/max/mean	min/max/mean	min/max/mean
Protein	46.41/244.27/87.61	46.86/155.04/77.64	72.57/195.09/113.01	66.23/264.00/173.8	73.63/155.85/101.42
Ligand	51.78/96.45/64.41	42.98/94.80/62.07			
R.m.s. deviations					
Bond lengths (Å)	0.003(0)	0.003(0)	0.003(0)	0.003(0)	0.003(0)
Bond angles (°)	0.498(0)	0.540(11)	0.521(0)	0.593(0)	0.527(0)
**Validation**					
MolProbity score	1.32	1.35	1.53	1,91	1.35
Clashscore	2.60	3.07	3.93	4.78	3.16
CaBLAM outliers (%)	2.29	1.92	1.76	4.08	1.26
Rotamer outliers (%)	0.59	0.76	1.45	1.61	1.36
Cβ outliers (%)	0	0	0	0	0
Ramachandran plot					
Favored (%)	96.05	96.3	96.52	91.42	97.22
Allowed (%)	3.87	3.67	3.36	8.58	2.74
Disallowed (%)	0.08	0.03	0.12	0.00	0.04

Table S6. Identification of LtPMF Complex III_2 subunits by cryo-EM, BN-PAGE complexome profiling, and comparative genomics, including residues assigned in the cryo-EM map, related to Figures 4 and 5. Subunits were identified through the integration of the cryo-EM map, BN-PAGE complexome profiling, and comparative genomics across multiple eukaryotic lineages, including *A. castellanii*, *A. thaliana*, *T. gondii*, *S. cerevisiae*, and *H. sapiens*.

Gene product	ID	Cryo-EM Structure	BN-PAGE Complexome	Homologs	Total residues	Assign residues
Heme protein	LtaP07.0060	•	•	•	258	1–258
MPP-β	LtaP35.1450	•	•	•	490	1–490
Ubiquinol-cytochrome c reductase complex 14 kda protein	LtaP35.0210	•	•	•	202	12–202
MPP-α	LtaP13.0770	•	•	•	527	49–527
Rieske	LtaP35.1590	•	•	•	297	19–297
Cytb	MT-CYB	•	•	•	371	1–371
Hypothetical protein, conserved	LtaP17.1410	•	•		67	1–67
Hypothetical protein, conserved	LtaP32.3800	•	•		147	1–147
Hypothetical protein, conserved	LtaP35.0250	•	•		119	13–119
Hypothetical protein, conserved	LtaP27.0110	•	•		86	11–85
Ubiquinol-cytochrome c reductase	LtaP31.3010	•	•	•	70	1–70

Table S7. Identification of LtPMF Complex IV_2 subunits by cryo-EM, BN-PAGE complexome profiling, and comparative genomics, including residues assigned in the cryo-EM map, related to Figures 4 and 6. Subunits were identified through the integration of the cryo-EM map, BN-PAGE complexome profiling, and comparative genomics across multiple eukaryotic lineages, including *A. castellanii*, *A. thaliana*, *T. gondii*, *S. cerevisiae*, and *H. sapiens*.

Gene_product	ID	Cryo-EM Structure	BN-PAGE Complexome	Homologs	Total residues	Assign residues
COX1	MT-COX1	•	•	•	549	1–549
COX2	MT-COX2	•	•	•	210	1–210
COX3	MT-COX3	•	•	•	287	1–287
Cytochrome C oxidase subunit VI, putative	LtaP21.2040	•	•	•	157	1–157
Cytochrome c oxidase assembly factor-like protein	LtaP05.0800	•	•		147	20–147
Hypothetical protein, conserved	LtaP04.0630	•	•		200	1–200
Hypothetical protein, conserved	LtaP09.0030	•	•		107	1–107
Cytochrome c oxidase subunit iv	LtaP12.0690	•	•		343	33–343
Hypothetical protein, conserved	LtaP23.0450	•	•		118	24–118
Cytochrome c oxidase VII, putative	LtaP25.1190	•	•		345	181–345
Cytochrome c oxidase subunit V, putative	LtaP26.1720	•	•		196	18–196
P27	LtaP28.1050	•	•		240	1–223
Cytochrome c oxidase VIII, putative	LtaP31.2040	•	•		279	153–279
Hypothetical protein, conserved	LtaP36.0470	•	•		225	24–225
Cytochrome c oxidase subunit I	LtaP36.7290	•	•		150	38–150
Hypothetical protein, conserved	LtaP33.2260	•	•		116	22–116
Hypothetical protein, conserved	LtaP36.1760	•	•		106	24–106

Table S8. Identification of LtPMF Complex V (ATP synthase) subunits by cryo-EM, BN-PAGE complexome profiling, and comparative genomics, including residues assigned in the cryo-EM map, related to Figures 4 and 7. Subunits were identified through the integration of the cryo-EM map, BN-PAGE complexome profiling, and comparative genomics across multiple eukaryotic lineages, including *A. castellanii*, *A. thaliana*, *T. gondii*, *S. cerevisiae*, and *H. sapiens*.

Gene_product	ID	Cryo-EM Structure	BN-PAGE Complexome	Homologs	Total residues	Assign residues
mitochondrial ATP synthase subunit *i/j*	LtaP03.0910	•	•		104	2–103
mitochondrial ATP synthase subunit *d*	LtaP08.0840	•	•		366	30–168, 185–323
mitochondrial ATP synthase subunit ATPTB3	LtaP11.1000	•	•		349	92–349
mitochondrial ATP synthase subunit ATPTB1	LtaP21.1790	•	•	•	384	1–384
mitochondrial ATP synthase subunit ATPEG3	LtaP22.1610	•	•		145	64–141
mitochondrial ATP synthase subunit *f*	LtaP25.1690	•	•		146	4–126
mitochondrial ATP synthase subunit ATPTB11	LtaP25.2210	•	•		156	20–155
mitochondrial ATP synthase subunit *k*	LtaP26.1030	•	•		200	96–199
mitochondrial ATP synthase subunit ATPTB6	LtaP27.0660	•	•		202	38–200
mitochondrial ATP synthase subunit 8	LtaP34.1060	•	•		122	39–122
mitochondrial ATP synthase subunit *b*	LtaP34.3980	•	•		104	30–104
mitochondrial ATP synthase subunit ATPTB12	LtaP08.0990	•	•		99	1–99
A6	MT-ATP6	•	•		232	1–231
mitochondrial ATP synthase subunit *e*	LtaP25.0620	•	•		148	61–119
Mitochondrial ATP synthase subunit c-1	LtaP24.0710	•	•	•	106	31–106
mitochondrial ATP synthase subunit *g*	LtaP33.3040	•	•		152	26–152
mitochondrial ATP synthase subunit ATPTB4	LtaP36.4590	•	•		160	24–160
mitochondrial ATP synthase subunit ATPEG4	LtaP27:632926-633111	•	•		62	5–59
